# Comprehensive study of excess phosphate response reveals ethylene mediated signaling that negatively regulates plant growth and development

**DOI:** 10.1038/s41598-017-03061-9

**Published:** 2017-06-08

**Authors:** Devesh Shukla, Claire A. Rinehart, Shivendra V. Sahi

**Affiliations:** Department of Biology, 1906 College Heights, Western Kentucky University, Bowling Green, 42101-1080 Kentucky, USA

## Abstract

Excess Phosphorus (P) in agriculture is causing serious environmental problems like eutrophication of lakes and rivers. Unlike the enormous information available for phosphate starvation response (P_0_), very few information is available for the effect of excess phosphate P_i_ on plants. Characterization of Excess Phosphate Response (EP_i_R) is essential for designing strategies to increase phosphate accumulation and tolerance. We show a significant modulation in the root developmental plasticity under the increasing supply of excess P_i_. An excess supply of 20 mM P_i_ (P_20_) produces a shallow root system architecture (RSA), reduces primary root growth, root apical meristem size, and meristematic activity in Arabidopsis. The inhibition of primary root growth and development is indeterminate in nature and caused by the decrease in number of meristematic cortical cells due to EP_i_R. Significant changes occurred in metal nutrients level due to excess P_i_ supply. A comparative microarray investigation of the EP_i_R response reveals a modulation in ethylene biosynthesis and signaling, metal ions deficiency response, and root development related genes. We used ethylene-insensitive or sensitive mutants to provide more evidence for ethylene-mediated signaling. A new role of EP_i_R in regulating the developmental responses of plants mediated by ethylene has been demonstrated.

## Introduction

Phosphate (P_i_) availability is often limited in the soil, because of its low mobility, thus creating a major problem in various regions of the world. Several studies have undertaken to characterize plant responses under P_i_ starvation condition^1–4^. On the other hand in the developed industrialized world, the excessive application of P_i_ based chemical fertilizers and phosphate-rich animal manures often results in the accumulation of P_i_ in the top soil^5–8^. It leaches into surface water runoffs posing serious environmental concerns like eutrophication^5–11^. Chesapeake Bay region Maryland, Okeechobee Basin region of Florida (USA) and Sussex County, Delaware (USA) were rated as excessive in phosphorus^8, 12, 13^. The maximum inorganic P content reaches up to the order of 2610–7343 mg kg^−1^ in the soil around the world^14–16^. However, the availability of P varies greatly in different soils of the different regions. In Mehlich 3 soil of New York state (USA), total inorganic phosphorus level reached as high as 7343 mg P kg^−1^, but the resin-extractable P (available phosphorus) was found up to 2330 mg P kg^−1^ ^15^. In the same soil, the content of dilute acid P_i_ pool increased up to 4815 mg kg^−1^ ^15^. In a recent report, swine manure amended paddy soil profile showed an orthophosphate level up to 2610 mg kg^−1^ with 80% extraction efficiency^16^. Moreover, this situation may increase as Tillman *et al*.^17^ predicted that the most natural ecosystems will be converted into agriculture land by the year of 2050 owing to the increase in demand for food. To mitigate the adverse effect of agriculture expansion, we need to explore modern biotechnological tools to increase phosphate acquisition efficiency of crop plants which require less input of fertilizer.

In our earlier studies, we have evaluated the potential of *Lolium multiflorum* cultivars for remediation of excess phosphorus^8, 10, 11^. Studies conducted to test the efficacy of the plant-based remediation supports the use of this technology; however, the current P uptake rates in plants are not sufficient enough to tackle the problem^8, 9^. There is a need to significantly increase the P uptake and accumulation efficiency in plant to achieve the goal^9^. Genetic engineering may hold the promise for developing P-hyperaccumulator plants. However, no study has been taken up to investigate the plant responses under a wide range of P regime in detail. Furthermore, unlike the well-documented mechanism of heavy metal toxicity^18^, there is no information available about the toxicity of phosphorus in plants except few reports showing Fe or Zn deficiency under high P condition^19, 20^. Therefore, to design an effective strategy for P acquisition, accumulation, and tolerance, we need to understand and characterize the plant responses under excess phosphate conditions.

Several adaptive responses to P_i_ starvation have been established; for example, attenuation of primary root growth, increase in the lateral root density, and accumulation of anthocyanin^21–24^. Several transcriptomic studies have been carried out in plants to characterize the molecular response to phosphate deficiency or sufficiency using the ATH1 microarray chips^25–28^. Unlike these enormous investigations on phosphate (P_i_) starvation, very few studies have been conducted to characterize plant responses under excess phosphate^10, 29^. A comprehensive morphophysiological study coupled with high-throughput gene expression technology to characterize the effect of varying concentrations of excess P_i_ on plants is still lacking. Thus we have designed experiments to augment the present understanding of the excess phosphate response (EP_i_R) in the root and shoot tissues of Arabidopsis. We also intend to unravel the underlying signaling mechanism that regulates growth and development of plant during excess phosphate treatment.

Here, we showed concentration dependent changes in root system architecture (RSA), total soluble P_i_ content, and metal content (P, Fe, Zn, Ca, Mn). The global transcriptomic profiling reveals that the excess P_i_ supply induces an ethylene-mediated response which may be responsible for the alteration of the phenotype. We further provide evidence for the involvement of ethylene by using ethylene-insensitive mutants (*etr1–3*, *ein4*, *ein2-T*) and constitutive ethylene sensitive mutant (*ctr1–1*).

## Results

### Excess P_i_ supply affects shoot morphophysiological traits

**Figure 1:**
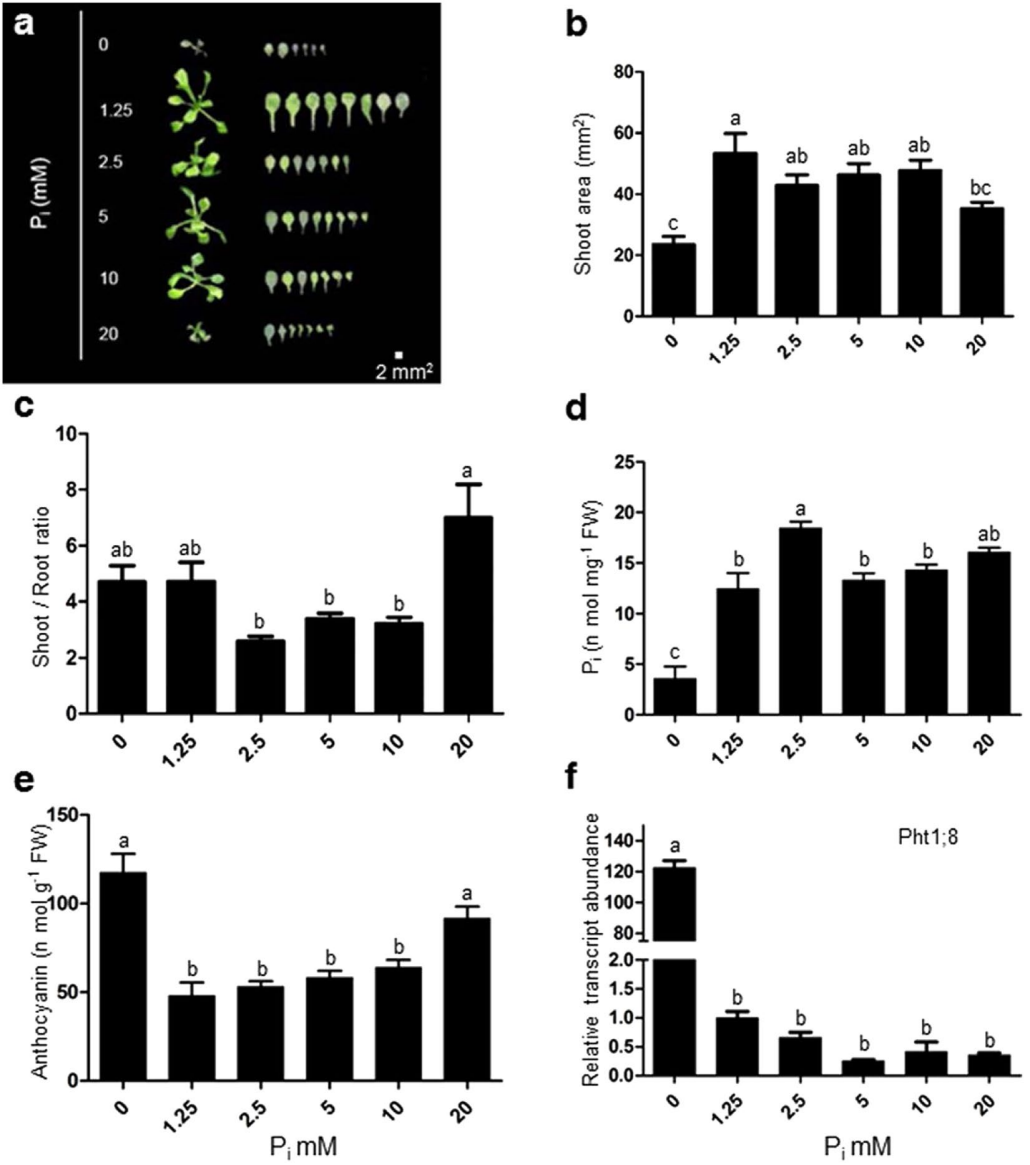
Supply of different concentrations of excess phosphate (P_i_) modulates morphophysiological traits of the shoot. WT seedlings were initially grown hydroponically in 0.5X MS media for 5 days thereafter subjected to different P_i_ supplies (0, 1.25, 2.5, 5, 10, 20 mM) and grown for 7 days. Seedlings were spread on the agar Petri-plates for measurement of traits (**a,b**). Data are presented for shoot area (**b**), shoot to root ratio (**c**), total soluble P_i_ content (**d**), anthocyanin content (**e**), the expression level of Pht1;8 in shoot (**f**). Values are means ± SE, *n* = 21 (**b,c**), *n* = 3 consists of 30–70 mg shoot tissue in each assay (**d**, and **e**), *n* = 3 consists of 50 mg shoot tissue for each replicate (**f**). Mean Bars with different alpha letter differ significantly (P ≤ 0.05) according to analysis of variance (1X-ANOVA) with Tukey’s Multiple Comparison Posttest.

**Figure 2:**
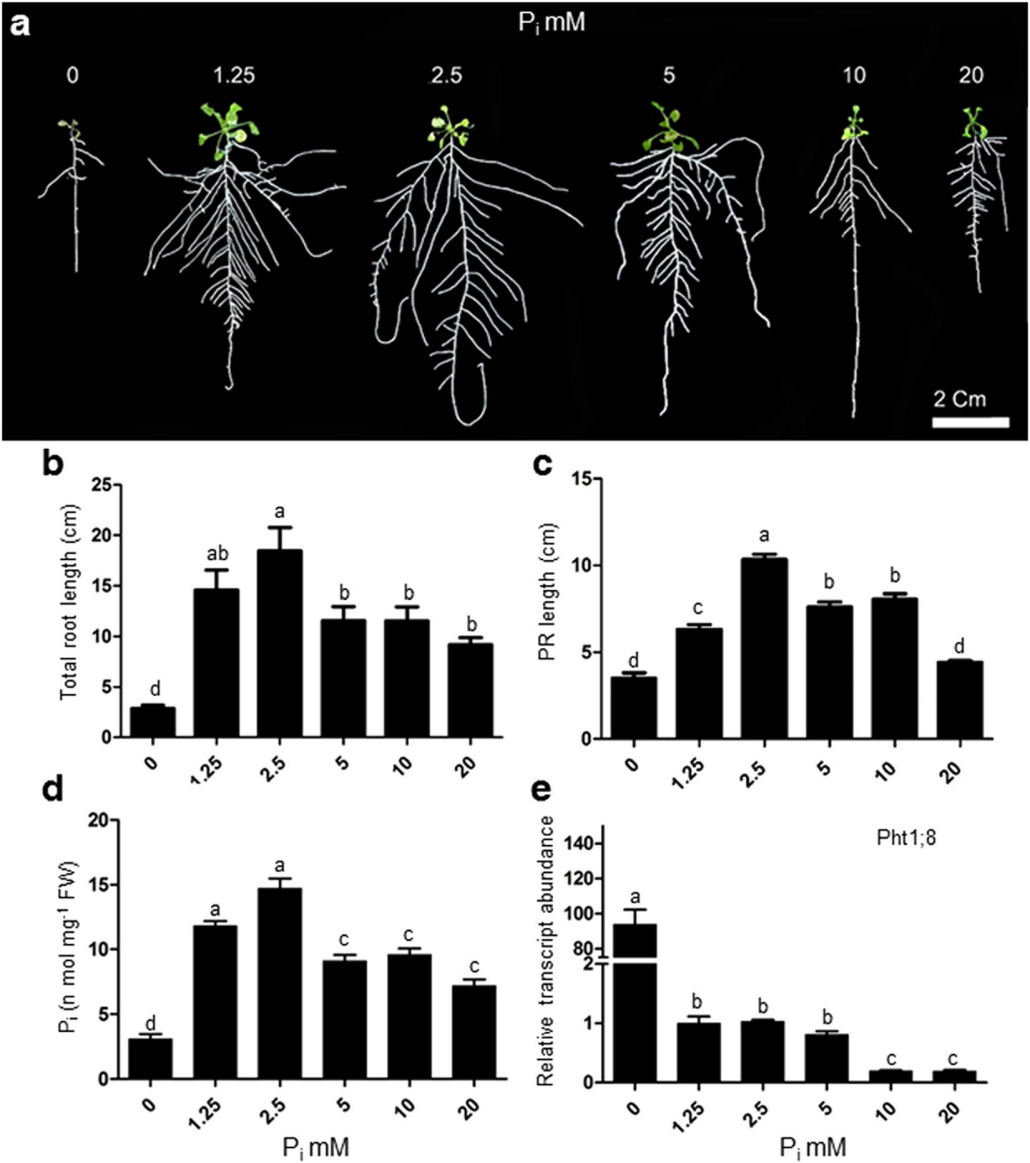
Supply of different concentrations of excess phosphate (P_i_) modulates different morphophysiological parameters of root. WT seedlings were grown as described in the caption to Fig. 1. Data are presented for Total root length (**b**), Primary root length (**c**), total soluble P_i_ content (**d**), Expression level of Pht1;8 (**e**), Values are means ± SE, and *n* = 21 (**b,c**) or *n* = 3 consists of 20–50 mg root tissue in each assay (**d**). *n* = 3 consists of 50 mg root tissue for each replicate (**e**). Mean Bars with different alpha letter differ significantly (P ≤ 0.05) according to analysis of variance (1X-ANOVA) with Tukey’s Multiple Comparison Posttest.

The morphophysiological traits of Arabidopsis were examined after 7 days of growth under P_0_ (0 mM), P_1.25_ (1.25 mM), P_2.5_ (2.5 mM), P_5_ (5.0 mM), P_10_ (10 mM) and P_20_ (20 mM) P_i_ concentrations (Figs 1 and 2). Treatments P_1.25_, P_2.5_, P_5_ and P_10_ exhibited a larger shoot area similar to each other (Fig. 1b). P_20_ showed a significant lower shoot area compared to P_1.25_ only (Fig. 1b). The P_0_ treatment showed a leaf area significantly smaller than all other treatments except P_20_. The shoot to root ratio increased significantly at the P_20_ as compared to P_10_, P_5_, and P_2.5_ treatments. It indicates that the shoot growth was not as much compromised as root at the high P_20_ treatment (Fig. 1c). The physiologically available total soluble P_i_ content peaked at P_2.5_ but later dropped to a plateau, at treatments P_5_, P_10_, and P_20_ (Fig. 1d). Anthocyanin content is considered to be a specific marker for the P_i_ deficiency response and was found to be highest at P_0_ and P_20_ (Fig. 1e). The expression level of Pht1 family transporters significantly repressed under the excess concentrations of P_i_ (Fig. 1f, Supplementary Fig. S1). Overall the shoot growth was promoted at P_1.25_ and adversely affected at P_0_ and P_20_ treatments.

### Excess P_i_ supply affects morphophysiological traits of root

The excess P_i_ treatment (P_20_) produced a striking root phenotype exhibiting shorter, shallow and less branched RSA (Fig. 2a). Total root length and primary root length increased till P_2.5_ and then decreased under subsequent concentrations with a maximum decrease at P_20_ (Fig. 2b,c). The root growth showed a correlation with total soluble P_i_ content in the root that was highest at P_2.5_ thereafter dropped to a plateau at treatments P_5_, P_10_, with a maximum decrease at P_20_ treatment (Fig. 2d). The soluble P_i_ content of shoot was higher as compared to root under different P_i_ treatments. It may be due to the complete repression of P_i_ uptake transporters expression like Pht1 family members in root (Fig. 2e, Supplementary Fig. S1); whereas, the expression of PHO1, a phosphate xylem loading transporter, was found to be increased at excess P_i_ concentrations (Supplementary Fig. S2).

### Excess P_i_ supply modulates the number and length of higher order lateral roots

**Figure 3:**
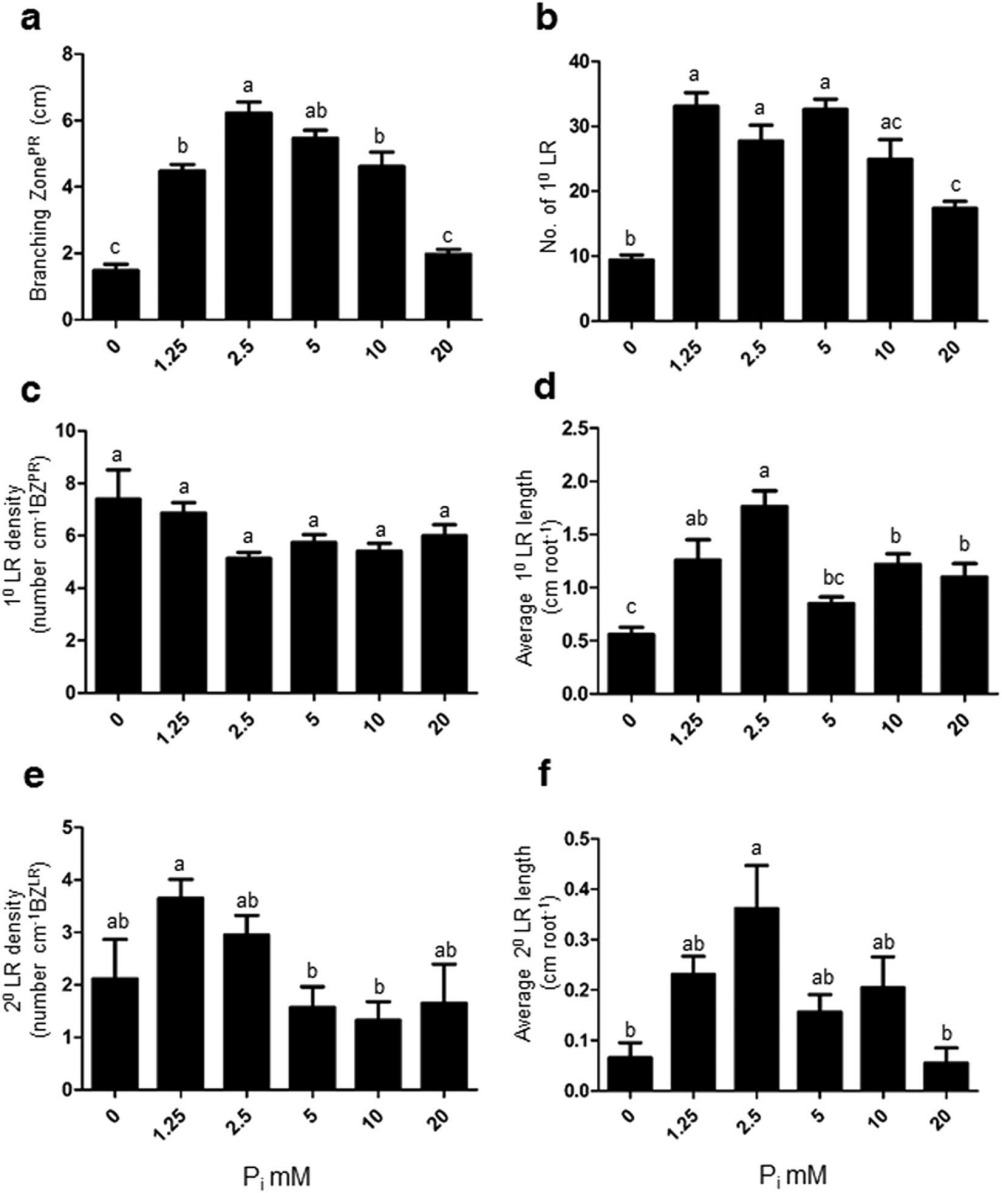
Supply of different concentrations of excess phosphate (P_i_) modulates different traits of root system architecture. WT seedlings were grown as described in the caption to Fig. 1. Individual seedlings were carefully pulled out from the mesh and spread on the agar Petri-plates for documentation of different traits (**a–f**). Data are presented for Branching zone (**a**), number of first order lateral root (1° LR) (**b**), first order lateral root density (1° LR density) (**c**), average first order lateral root length (1° LR length) (**d**), Second order lateral root density (2° LR density) (**e**), average second order lateral root length (2° LR length) (**f**). Values are means ± SE, and *n* = 21 (**a–f**). Mean Bars with different alpha letter differ significantly (P0 ≤ 0.05) according to analysis of variance (1X-ANOVA) with Tukey’s Multiple Comparison Posttest.

The length of the branching zone (BZ^PR^) increased till treatment P_2.5_ thereafter decreased at P_5_ and P_10_ and then showed a sharp decline at P_20_ (Fig. 3a). The number of primary lateral roots (1° LR) did not significantly change from P_1.25_ to P_10_ but later significantly decreased at P_20_ (Fig. 3b). The primary lateral root density did not change significantly at any of the concentration of treatments (P_0_ to P_20_) (Fig. 3c). The average 1° LR length increased till the treatment of P_2.5_ thereafter decreased at subsequent treatments but maintained a level found similar to P_1.25_ (Fig. 3d). The 2° LR density significantly decreased at P_5_, and P_10_ treatments as compared to P_1.25_ and rest of the treatments did not show any significant difference (Fig. 3e). Average 2° LR length significantly increased at P_2.5_ as compared to treatments P_0_ and P_20_ and remaining there was no significant difference among the treatments (Fig. 3f). Altogether these results show the initial P_i_ concentrations till 2.5 mM P_i_ promotes the root growth but subsequent concentrations of excess phosphate (10 or 20 mM) showed an adverse effect on RSA traits.

### Excess P_i_ supply inhibits primary root growth by attenuating meristematic activity

**Figure 4:**
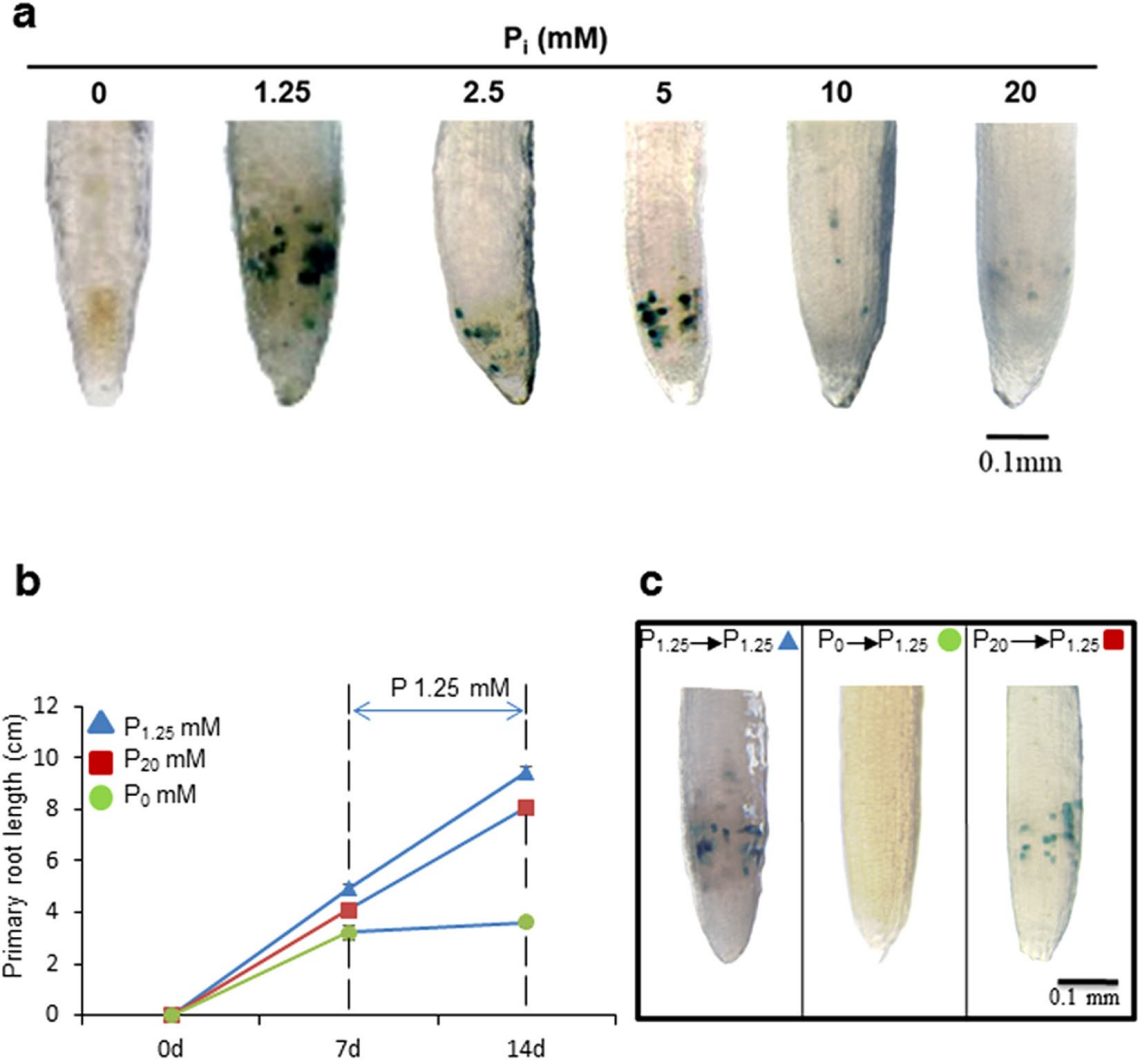
Excess P_i_ treatment attenuates the meristematic activity of primary root indeterminately. Histochemical GUS staining of primary root tips of *CycB1*;*1:uidA* transgenic seedlings grown as described in the legend to Fig. 1 (**a**). WT Arabidopsis seedlings were grown on P_1.25_, P_0_, and P_20_ media for 7 days as described earlier, thereafter replenished with P_1.25_ media and growth monitored after 7 days (**b**). A similar experiment was performed with transgenic seedlings of *CycB1;1:uidA* and documented for histochemical GUS staining (**c**). Pictures are representative of 10–12 seedlings. Values are means ± SE; *n* = 21 (**b**).

Earlier reports demonstrated a loss of apical meristem activity of primary root during P_i_ deprivation which results in a determinate developmental shift^30, 31^. Primary root tip of seedlings grown under the treatment of P_1.25_, P_2.5_, and P_5_, showed a high staining of *CycB1;1::uidA* activity; however, a significant decline in staining was observed under P_10_ and P_20_, treatment respectively (Fig. 4a). It suggests that the higher concentrations of P_i_ supply trigger the loss of meristematic activity. The next question is whether this developmental shift is determinate or indeterminate. To answer this, we transferred the plants grown in P_1.25_, P_20_ and P_0_ medium to P_1.25_ medium and allowed them to grow further for 7 days. Interestingly, the length of primary root (P_20_, P_1.25_) increased nearly 2-fold at 14 day relative to earlier time point (7 day); whereas the length of plants grown in P_0_ did not gain any significant increase under the same conditions (Fig. 4b,c). Similar parallel experiment was setup with *CycB1;1::uidA* transgenic plants to record the meristematic activity. Plants replenished into P_1.25_, P_20_ showed GUS staining, and plants replenished into P_0_ did not show any GUS staining (Fig. 4c). It suggests that inhibition of primary root growth by P_20_ is developmentally indeterminate in nature.

### Evaluation of metal nutrients contents under excess P_i_ supply

**Figure 5:**
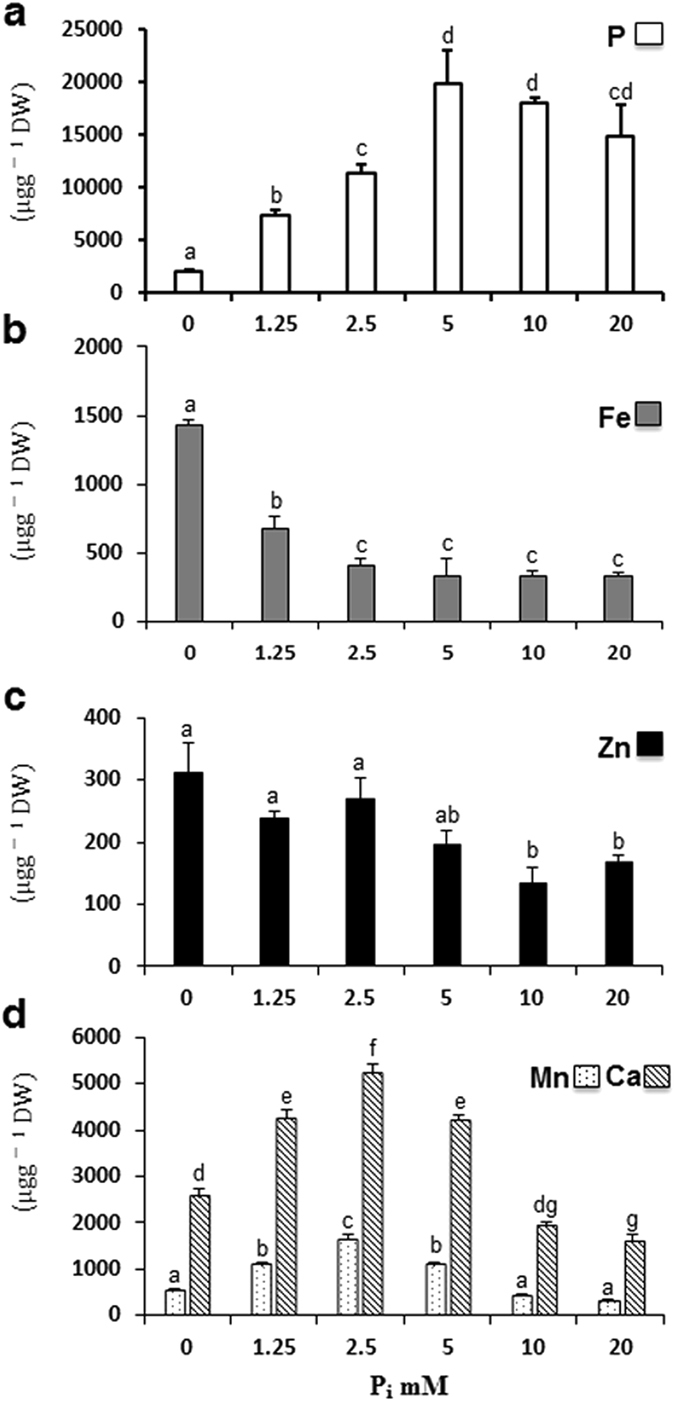
Metal nutrients analysis of Arabidopsis seedlings grown on varying concentrations of P_i_ hydroponically as described in the legend to Fig. 1. Data are presented as phosphorus content (**a**), iron content (**b**), zinc content (**c**), manganese and calcium content (**d**). Values are means ± SE; n = 3 replicates of 25 mg of dry weight of seedlings tissue. Mean Bars with different alpha letter differ significantly (P ≤ 0.05) according to analysis of variance (1X-ANOVA) with Tukey’s Multiple Comparison Posttest.

We estimated the level of P, Fe, Zn, Ca, and Mn in Arabidopsis seedlings grown under excess P_i_ using ICP-OES. A concentration dependent increase was observed in P content till P_5_; thereafter a steady level was observed at P_10_ and P_20_ (Fig. 5a). In contrast, the maximum level of Fe was found at P_0_ that rapidly depleted in the presence of P_i_ until P_2.5_ and then maintained a steady level at subsequent concentrations (Fig. 5b). Zn content did not change significantly at increasing concentration of P_i_ till P_5_, thereafter only a significant decrease was observed at P_10_ and P_20_ (Fig. 5c). The content of Ca and Mn peaked at P_2.5_ then begin to drop at the subsequent treatments (Fig. 5d). Overall, the higher concentrations of P_i_ adversely affect the metal nutrients contents.

### Transcriptomic comparison of excess phosphate response (EP_i_R) with phosphate starvation response (P_i_SR)

**Figure 6:**
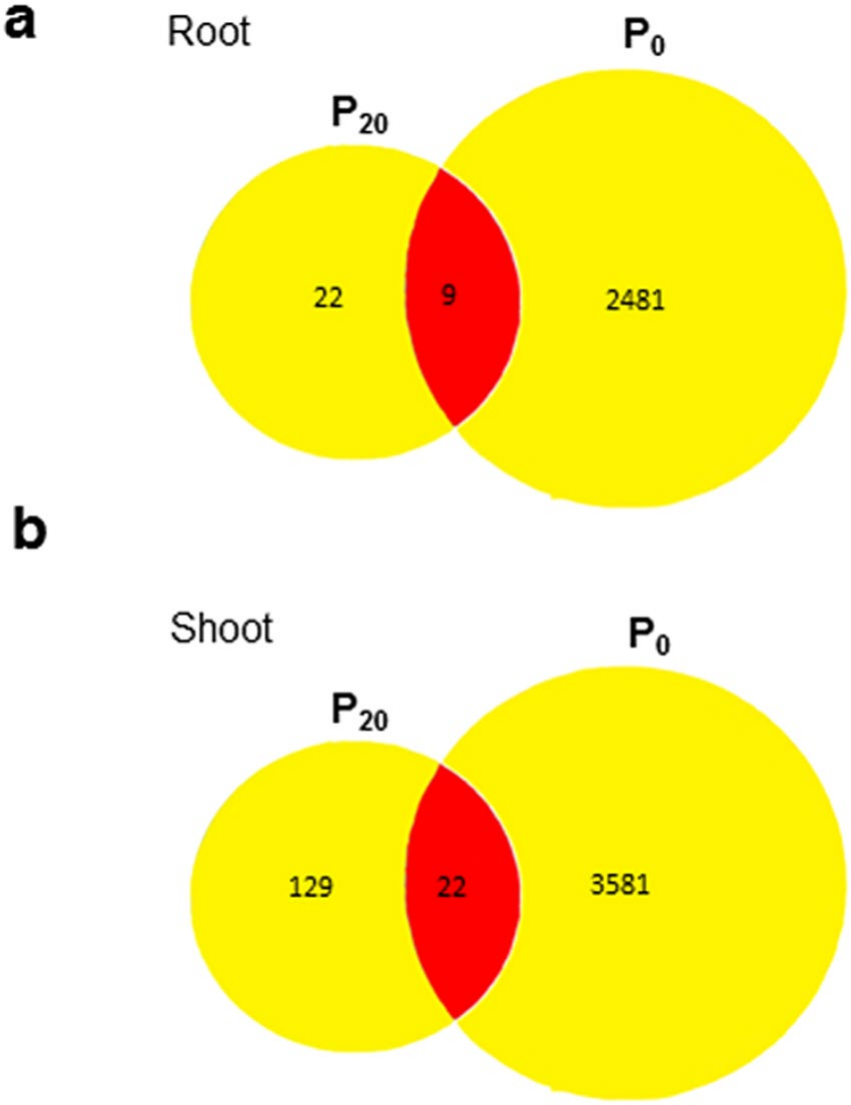
Differentially regulated genes, due to treatments P_20_ (20 mM P_i_) or P_0_ (no P_i_) relative to control (1.25 mM P_i_) in root and shoot. Venn diagrams show the total number of uniquely and commonly regulated genes due to treatments P_0_ and P_20_ in root (**a**) and shoot (**b**).

We comparatively studied plant transcriptome under P_20_ and P_0_ supply. A novel unbiased exon ST array chip from Affymetrix microarray technology was used to hybridize the RNA isolated from root and shoot tissues of Arabidopsis plants treated with P_20_, P_0_ and, P_1.25_ (control). This array uses the entire transcript to measure the expression of a gene thereby generates a precise and unbiased gene expression data (Supplementary Dataset File 1). Principal component analysis revealed the differential responses between root and shoot tissues across the treatments (Supplementary Fig. S3). Treatment components, P_20_ and P_1.25_, were relatively close to each other, indicating some similarities in their transcriptomic profiles (Supplementary Fig. S3). We obtained a total number of 31 and 2490 differentially regulated transcripts due to treatment P_20_ and P_0_, respectively in root (Fig. 6a). In shoot, a total number of 151 and 3603 transcripts were found to be differentially regulated due to treatment P_20_ and P_0_, respectively (Fig. 6b). We found a greater number of differentially regulated genes in shoots compared to root. Shoot serves as a sink organ for phosphate and exhibits a systemic adaptive response under varying P_i_ availability. Since our emphasis on excess phosphate response, we annotated in detail those transcripts which were relevant to EP_i_R. In root, only 9 genes were co-regulated by P_20_, P_0_ treatments (Supplementary Dataset File 2). The flowering promoting factor 1 (FPF1) is expressed in response to gibberellin stimulus and implicated in root growth inhibition^32^. The NRT1.8 is a low-affinity nitrogen transporter removes N from xylem tissue^33^. Nitrogen uptake is reported to be regulated by varying P availability^34^. FRD3 (Ferric Reductase Defective 3) is an antiporter/efflux transporter encodes a member of MATE (Multidrug and toxin efflux) is expressed in roots but not in shoots^35^. Expansin A17, a member of the α-expansin gene family, causes loosening, and extension of plant cell walls and is involved in an ethylene mediated signaling pathway^36^. There were 22 transcripts uniquely regulated by P_20_ treatment in root (Supplementary Dataset File 2). These transcripts represent a significant enriched GO term, transition metal ion binding indicating metal nutrient deficiency response (Supplementary Dataset File 3, Supplementary Fig. S4). Nicotianamine synthase 2 (NAS2) is responsible for the synthesis of nicotianamine, involved in iron sensing and transport^37^. Ferric reduction oxidase 5, is reported to participate in iron reduction to facilitate the transport^38^. An unknown, defensin-like protein 203 was found to be highly expressed (33-fold) possessing a GASA-like (Gibberellin-regulated protein) domain in polypeptide sequence. We also found a novel upregulated calmodulin binding protein (FDR 0.25), an MLO14-like protein that possesses a 7-transmembrane domain similar to G-Protein Coupled Receptor (GPCR), known to express during seedling stage in developing primary root tip^39^ (Supplementary Dataset File 1).

In shoot, 22 transcripts were commonly regulated by both the treatments (Supplementary Dataset File 4). Three different peroxidase genes related to oxidative stress were found. Exordium (EXO//Phosphate-Responsive Family Protein), is a brassinosteroid stimulus-response gene, localized in the cell wall, was found to be upregulated^40^. These transcripts were associated with GO terms like lipid biosynthetic process and hydrolase activity (Supplementary Dataset File 3, Supplementary Fig. S5). Two other important genes (FDR ≤ 0.28) co-regulated were ACS7 and MDR4 (Supplementary Dataset File 1). ACS7 is an important member of ethylene biosynthetic pathway. MDR4 is an auxin influx transporter mediates basipetal transport of auxin in hypocotyl and root tip^41^. A number of 129 transcripts were uniquely regulated in shoot, and some of the important ones are described here. The AtACS11 gene, involved in ethylene biosynthesis, was found to be up-regulated. Four ethylene responsive factors namely ERF003, ERF012, ERF022, and ERF055, were found to be expressed. ATPMEPCRB is a probable pectin esterase inhibitor was found to be expressed. A recent report demonstrates that the pectin content plays an active role in solubilizing phosphate in the cell wall^42^. These transcripts represent enriched GO terms such as root system development, mitochondrial organization, protein targeting, RNA methylation, nucleotide metabolic process and response to metal ion (Supplementary Dataset File 3, Supplementary Dataset File 4, Supplementary Fig. S6).

**Figure 7:**
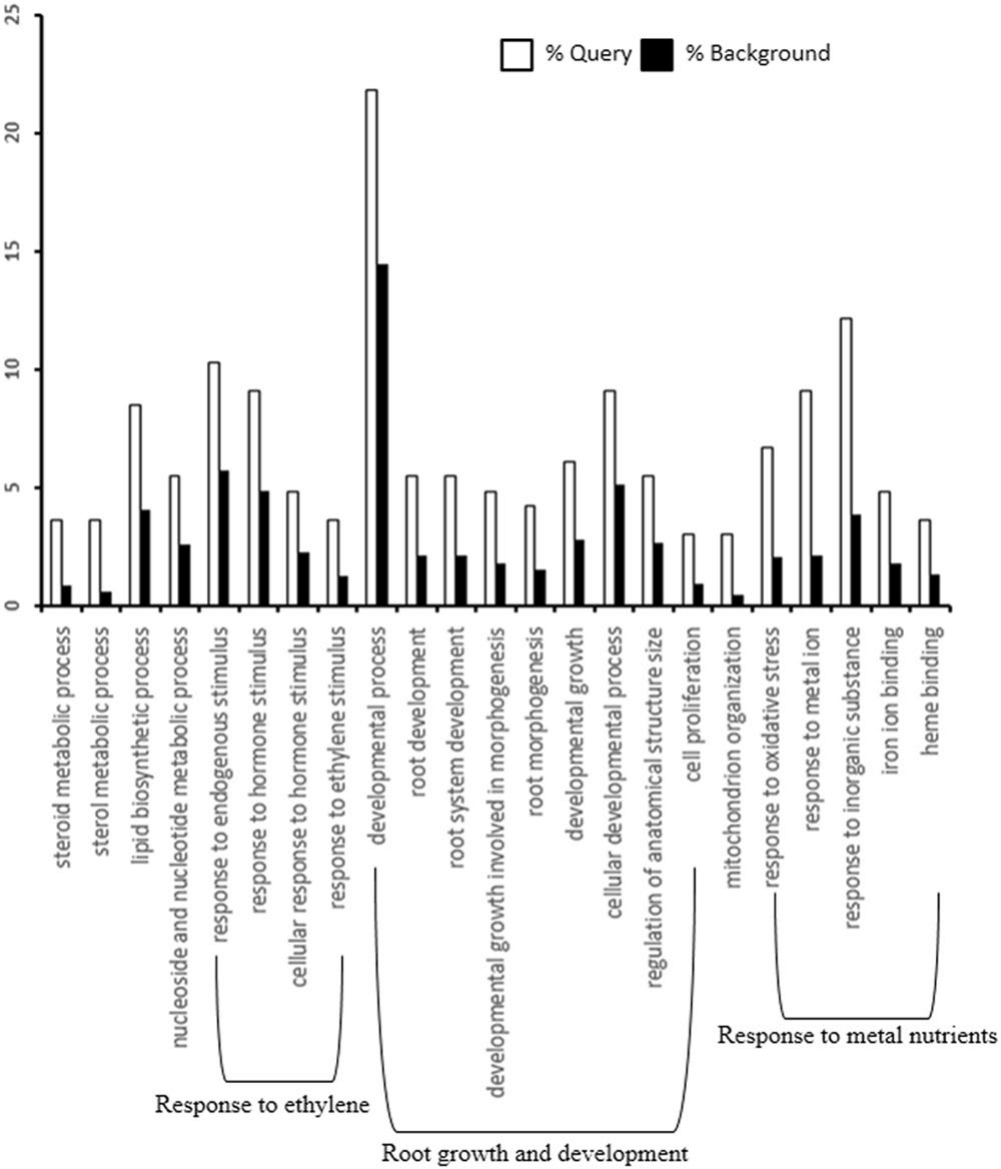
Singular Enrichment Analysis (SEA) for differentially expressed genes of root + shoot due to P_20_ (20 mM) treatment. It shows the change in ethylene, root growth and development, and response to metal nutrients related processes. The analysis was performed using the agriGO analysis tool under advanced mode, Fisher test with P < 0.05 and minimum 5 mapping entries. The white bar shows the fraction of query genes, and the black bar shows a fraction of genes in background (whole genome).

To obtain a picture of the whole plant as a system, we combined root and shoot transcripts differentially expressed under excess phosphate (P_20_) regime and performed GO enrichment analysis. We observed three distinct biological processes enriched such as response to ethylene stimulus, root growth and development and response to metal nutrients (Fig. 7, Supplementary Fig. S7, Supplementary Dataset File 3). It provides evidence that the plant as a whole induces ethylene mediated signaling when incubated under excess phosphate regime. Altogether, these results indicate the underlying signaling mechanism which involves ethylene during excess phosphate response.

### Validation of microarray expression data through qRT-PCR

Sixteen key genes were selected for validation of microarray data on the basis of metal ion deficiency response, ethylene mediated signaling, and root development (Supplementary Fig. S8). The expression of selected genes is more or less consistent with the microarray results. In general, treatment specific expression of genes is more consistent with the microarray results compared to tissue-specific expression. The iron deficiency response has been validated in root under P_20_; whereas, some members (NAS2, FRO5) showed deficiency response in shoot even under P_0_. It may be due to the variation in availability of different metal cations among the root and shoot. Ethylene biosynthesis and signaling related responses were validated; although, root and shoot were more or less equally responsive to both P_i_ treatments except ERF003. ERF003 showed a differential expression specifically under P_20_ in both tissues. Root development related genes also followed a similar expression trend with the array result; however, MLO14 was distinctly induced in root by P_20_. DEFL203 followed more or less similar trend as MLO14. Two oxidative stress related genes showed a high expression in root and shoot tissues under P_20_. Differential expression was confirmed for a SPX domain-containing protein that has been implicated in the P_i_ starvation response^4^. Overall, the expression of these genes coincides with array results.

### Ethylene insensitive mutants show a better developmental response under excess phosphate

**Figure 8:**
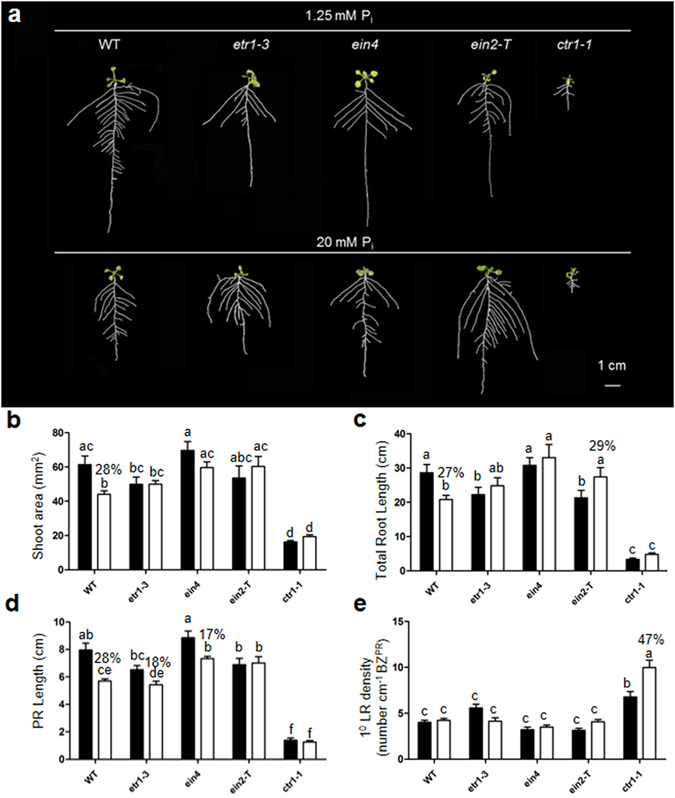
Root system architecture of wild type, ethylene-insensitive mutants (*etr1–3*, *ein4*, *ein2-T*) and sensitive mutants (*ctr1–1*) under 1.25 mM P_i_ and 20 mM P_i_. Wild-type (WT) and ethylene mutants were grown as described in the caption to Fig. 1. Individual seedlings were spread as described in the caption to Fig. 3 for documentation of different traits (**b–e**). Data are presented for shoot area (**b**), total root length (**c**), primary root length (**d**), and 1° lateral root density (**e**). Black and white bar represent treatments 1.25 mM and 20 mM, respectively. Values are means ± SE *n* = 15. Mean bars with different alpha letter differ significantly (P ≤ 0.05–0.001) according to two-way ANOVA with Bonferroni correction posttest comparisons.

To obtain more evidence for ethylene mediated signaling, we quantified shoot area and traits of RSA in ethylene-insensitive mutants *etr1–3*, *ein4*, *ein2-T* ^43, 44^, and a sensitive ethylene mutant *ctr1–1* ^45^ under control (P_1.25_) and excess P_i_ condition (P_20_) (Fig. 8). Shoot area decreased significantly by 28% in WT under P_20_. In the ethylene-insensitive mutants, no significant difference was observed in shoot area due to P_20_ treatment (Fig. 8b). Total root length of WT decreased significantly by 27% due to P_20_; whereas, it increased significantly by 29% in *ein2-T* or increased slightly in other insensitive mutants (Fig. 8c). Inhibition of primary root length was higher in WT than in ethylene-insensitive mutants *etr1–3*, *ein4*, *ein2-T*, (Fig. 8d). There was no significant difference in the phenotype of ethylene sensitive mutant *ctr1–1* except a significant increase in 1° LR density due to P_20_ (Fig. 8e). Altogether ethylene-insensitive mutants exhibited a better growth and development at P_20_ indicating some involvement of ethylene signaling.

### Excess phosphate response reduces the root apical meristem size by decreasing the cell number

**Figure 9:**
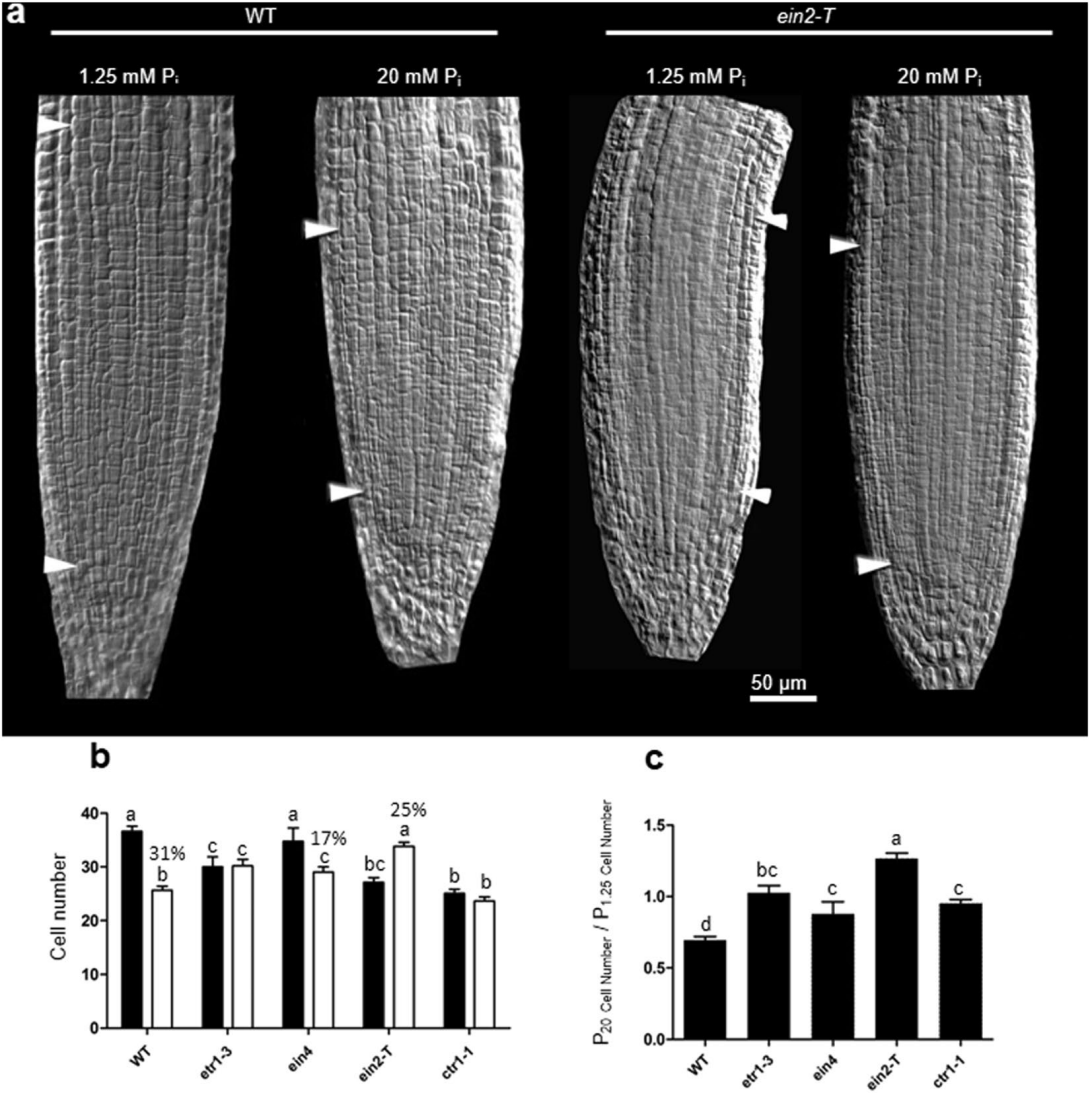
Excess phosphate supply reduced root apical meristem size of WT but not in ethylene- insensitive mutants. WT and ethylene insensitive (*etr1–3*, *ein4*, *ein2-T*) or sensitive mutant (*ctr1–1*) were grown as described in the caption to Fig. 1. A 5 mm section from the tip of primary root was cut and mounted on a slide with diluted Visikol in 10% glycerol, and root cells were visualized under the DIC filter. Root meristem size was measured by counting the number of meristematic cortex cells between the quiescent center (QC) and the first elongated cell. White arrows mark the QC and first elongated cortex cells in a representative picture of WT and *ein2-T* mutant (**a**). Data are presented for the number of cortical cells (**b**), and the ratio of RAM size (cell number) of plants grown at P_20_ to P_1.25_ conditions, as shown in (**c**). Values are means ± SE, and *n* = 15 (**b,c**). Bars with different alpha letter differ significantly (P ≤ 0.05–0.001) according to two-way ANOVA (**b**), and one-way ANOVA (**c**) with Bonferroni’s posttest.

To determine the nature of the primary root length reduction, we analyzed the size of root apical meristem (RAM) (Fig. 9a)^46^. RAM size decreased significantly in WT; whereas, it significantly increased in *ein2-T* or showed no change in other mutants due to P_20_ (Fig. 9b, Supplementary Fig. S9). No significant change in RAM size of *ctr1–1* mutant was observed due to P_20_ (Supplementary Fig. S10). We also analyzed the relative change in RAM size by taking the ratio at P_20_ to the P_1.25_. The relative increase in RAM size due to P_20_ treatment was significantly higher for ethylene-insensitive mutants as compared to WT (Fig. 9c). Taken together, it indicates the participation of ethylene mediated signaling during excess P_i_ treatment.

**Figure 10:**
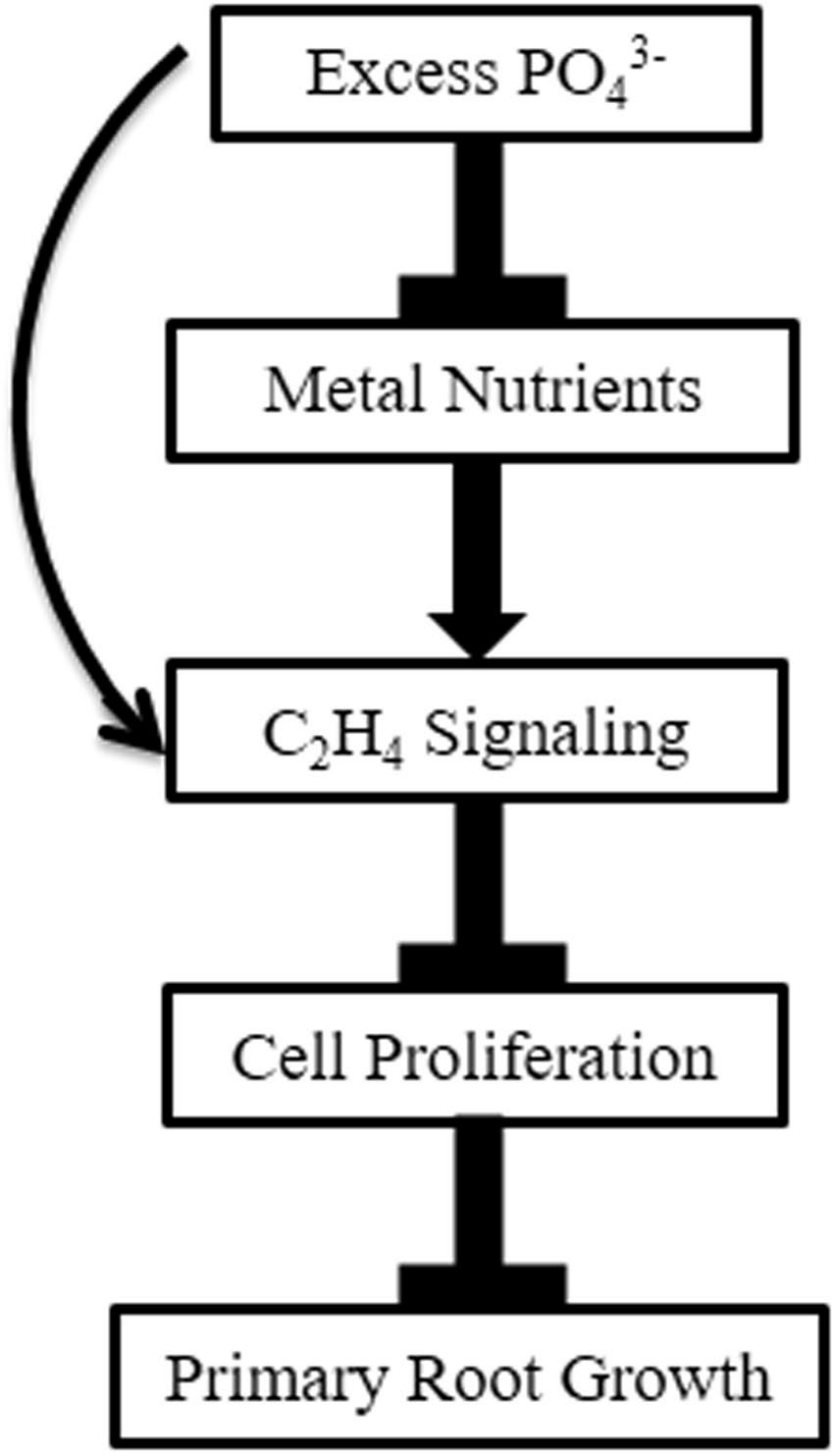
A common minimum consensus model displaying underlying signaling occurred during Excess Phosphate Response (EP_i_R). Excess phosphate treatment triggers the ethylene response may be directly or by altering nutrient profile and negatively regulates primary root growth by reducing cell proliferation in root apical meristem.

To summarize the important findings of this study, we proposed a schematic model which shows that the excess phosphate treatment induces the ethylene response may be directly or by altering the nutrient level and attenuates primary root growth by reducing the cell number in root apical meristem (Fig. 10).

## Discussion

Despite a rapid change in agronomic condition, causes environmental problems^17^, excess phosphate response in plants has not been elucidated in detail. Earlier reports showed that the high P condition in soil creates Fe and Zn deficiency in plants^19, 20, 47, 48^. In our earlier studies, we have attempted to identify genes modulated in crop plants like sunflower in excess P_i_ condition but obtained a little information^29^. In the present study, using morphophysiological, biochemical, genomics and genetic approaches we have characterized the excess phosphate response in Arabidopsis that indicates an ethylene mediated response.

We have shown that how varying concentrations of P_i_ could be sensed and rendered into phenotypic alterations. Different morphometric traits suggest that root morphology is more sensitive than shoot morphology by the supply of excess P_i_ (Figs 1 and 2). The total soluble P_i_ content was higher in the shoot under the excess P_i_ treatment points as compared to root (Figs 1d and 2d). The lesser level of soluble P_i_ in root may be linked to the shutdown of phosphate uptake transporters (Fig. 2e, Supplementary Fig. S1). At the same time, the mobilization of P_i_ from root to shoot keeps occurring because the expression of PHO1 was high (Supplementary Fig. S2a) therefore, leading to a low P_i_ level in the root (Supplementary Fig. S2b). The higher anthocyanin content of seedlings grown at P_20_, which was comparable to the level found in P_0_, supports this notion (Fig. 1e).

We observed a dramatic change in RSA by supplying varying concentrations of P_i_ (Fig. 2a). The primary root length increased initially and later decreased under increasing concentrations of P_i_ (Fig. 2c). This is in agreement with a study in which primary root length was shown to be inhibited by higher treatment concentrations of P_i_ at 10 and 25 mM^23^. The number of lateral roots remained steady till P_5_, then slightly decreased at P_10_ and with a significant decrease at P_20_ (Fig. 3b). This is consistent with an earlier study showing a sharp decline in the number of lateral roots at 10 and 25 mM P_i_^23^. Similarly, they showed a sharp decrease in L.R. density between 1 and 25 mM P_i_^23^. However, we did not observe any difference in L.R. density under P_0_, P_1.25_, and P_20_ (Fig. 3c). There are several contrasting reports on how P_i_ starvation-induced changes in number, length, and density of lateral roots. For example, an increase in L.R. density was reported under P_0_ condition^21, 23^. In contrast, a decrease in L.R. density under P_0_ conditions was also reported^24, 49, 50^. These variations may be due to the use of different growth conditions such as light, sucrose, incubation time^51, 52^. Also, the concentration of P_i_^23, 24^ and, the use of the various gelling media possessing varying levels of P_i_ contamination may attribute to these variations^52, 53^. Ethylene is known to reduce primary root length under P_i_-sufficient condition^54–56^. Ethylene was also reported to be involved in lateral root spacing and to negatively regulate the number of lateral roots while increasing the length of existing lateral roots^56^. Interestingly, we have observed attenuation of primary root length, reduction in the number of lateral roots, and no alteration in the length of lateral roots under excess P_10_ and P_20_ condition.

We observed a significant decline in *CycB1;1:uidA* staining in the primary root tip grown at P_10_ and P_20_ (Fig. 4a). More than one factor may be responsible for these results. One of the factors may be that a depletion of total physiologically available P_i_ which plays a critical role in the growth and development of root (Fig. 2d). Other regulating factors may be linked hormonal regulation. For example, indoleacetic acid (IAA) treatment inhibits primary root growth without affecting the meristematic activity, but since we observed a decline in the meristematic activity, auxin-mediated inhibition of primary root growth might be ruled out. Another hormone factor is ethylene which not only negatively-regulates primary root growth but may also be involved in repressing the Cyclin B1 activity^46^. Moreover, the inhibition of primary root growth and meristematic activity due to P_20_ was indeterminate thus developmentally distinct from the inhibition caused by phosphate starvation (P_0_) (Fig. 4b and c).

The nutrient composition of P, Fe, Zn, Mn and Ca plays a crucial role in determining RSA plasticity^24, 53^. Earlier studies reported that the micronutrients deficiency in plants caused by high P condition^19, 20, 47, 48^. We observed a maximum reduction in primary root under P_0_ and P_20_ however, developmental program were different. It is known that the loss of root meristematic activity under P_0_ condition is due to the elevated Fe level as it could produce hydroxyl radicals leading to the death of meristematic cells^47, 57, 58^ (Fig. 5b). Under P_20_, Fe level is relatively low, despite we observed a reduction in primary root length and root meristematic activity (Fig. 5b). It may be due to the depletion of other essential nutrients (Zn, Mn, Ca) in the seedlings (Fig. 5c,d). The low Fe content could also be a reason for an indeterminate reduction in root meristematic activity under P_20_ (Fig. 5b). Zn deficiency reduces the primary root length and enhances the lateral root number, but meristematic activity is not affected^53^. Ca deficiency also causes a decline in primary root elongation^24^. Taken together, it is plausible that the alteration in the level of nutrients elicits the hormonal responses that control the RSA plasticity^59^. Although, the specific coordination mechanisms involved in root development are not known.

The use of the Arabidopsis ST array offers access to an extensive number (28403) of transcripts relative to ATH1 array (22810 probes) and highlights the advantage of using 1.0 ST array chips. Eighteen tissue and treatment specific arrays were analyzed with the goal of creating a reference dataset to gain novel insight into the excess phosphate response (EP_i_R). We presented a comparative gene expression Venn diagram by distributing differentially regulated genes under EP_i_R, P_i_SR and both for root and shoot tissues as shown in Fig. 6. The majority of the differentially expressed genes and associated enriched GO terms under excess phosphate point towards ethylene mediated signaling (Fig. 7).

The gene AtACS11 and AtACS7 which is an isoform of 1-aminocyclopropane -1-carboxylate synthase (ACS) provides the rate-limiting step in ethylene biosynthesis were found to be upregulated^60^. We obtained four members of ERFs, expressed uniquely in the P_20_ treatment in the shoot (Supplementary Dataset File 4). AtERF003 and AtERF012 each have a repressor motif called EAR (ERF-associated amphiphilic repression) at the C-terminal. This motif is responsible for the repression of transcription of several target genes^61, 62^ and controls the major phenotypic changes in plant growth and development^63^. However, direct evidence is lacking for the function of AtERF003. Another gene Expansin A17, reported to be involved in ethylene signaling, was upregulated by P_20_ treatment and downregulated by P_0_^36^. Upregulation of several ethylene-related genes and their GO enrichment analysis suggest that ethylene at large mediating the excess phosphate response.

Nicotianamine synthase (NAS2), Ferric Reduction Oxidase 5 (FRO5), and FRD3 (Ferric Reductase Defective 3) were upregulated in root by P_20_ (Supplementary Dataset File 2). The expression of these genes is regulated by ethylene and was reported to be expressed under Fe, Zn and Cu deficiency^35, 37, 38, 64^ (Supplementary Dataset File 2). Some members of the peroxidase gene family were found to be differentially regulated by P_20_ and may be an indication of oxidative stress caused by metal deficiency (Supplementary Dataset File 2). Additionally, the GO enrichment analysis in root tissue resulted in GO terms like transition metal ion binding, response to metal ion, and heme binding (Supplementary Fig. 4). It suggests a metal ion deficiency occurring under excess phosphate condition.

We identified several enriched GO terms associated with root system development mainly for transcripts regulated in shoot (Supplementary Fig 6, Supplementary Dataset File 3). It indicates the important systemic role played by shoot regulating RSA under varying phosphate regime. Several previously known genes for controlling the RSA were identified. We found an upregulation of MLO 14 like protein in root, a recent study of MLO mutant revealed its involvement in polar auxin transport which is one of the components jointly responsible for an aberrant root phenotype^65^. A channeling protein, Annexin (ANNAT7) is reported to be involved in lateral root development by modulating the Ca^2+^ ion gradient^66, 67^. A defensin-like protein, DEFL203, was expressed at its maximum level in the P_20_ treatment. Defensins are small secreted peptides having diverse biological roles and may be involved in growth and development^68^. DEFL203 possesses a GARE element in its promoter region and a GASA-like domain in its peptide sequence that has a role in meristematic cell division activity^69^. The AtERF003 might play a major role in restructuring the root system. It is known to be involved in ethylene and/or gibberellic acid mediated signaling pathways^70^. The direct role of ERF in restructuring the root system architecture has been demonstrated in a study, in which the overexpression of AtERF070 leads to decrease in primary root length and lateral root number^71^.

Root system architecture is a complex trait as its development depends upon the hormonal interplay (ethylene, auxins, gibberellins, brassinosteroids and many more) and their interaction with the environment^72^. Since we obtained a set of genes which directly or indirectly points towards ethylene and signaling, we tested the hypothesis of ethylene by examining ethylene insensitive or sensitive mutants. Interestingly we observed a better growth phenotype in ethylene-insensitive mutants relative to WT under excess P_i_ condition (Fig. 8a). Constitutive ethylene sensitive mutant *ctr1–1* produces no significant change in phenotype presumably due to exhibiting a full response at control treatment in our experimental condition thus only a slight alteration in RSA occurred due to P_20_. Shoot area shows a better development in ethylene-insensitive mutants (Fig. 8b). There are reports which show the role of ethylene in negative regulation of shoot growth and development^73^. Primary root shows a relatively better growth phenotype in ethylene-insensitive mutants (Fig. 8d). We observed some variation in phenotype of *etr1–3*, *ein4*, and *ein2-T* relative to WT at control condition (P_1.25_) (Fig. 8). It may be due to the variation in responses depending upon the degree of ethylene insensitivity. For example, *ein4* mutant does not exhibit a strong insensitivity to ethylene rather behaves similar to WT^74^. Ethylene-sensitive mutant *ctr1–1* shows lesser growth relative to WT under normal growth condition^75^. We observed a significant increase in the 1° lateral root density of *ctr1–1* due to P_20_. This increase could be attributed to the increase in the number of lateral roots in a highly short primary root length (Fig. 8a).

Ethylene reduces root apical meristem (RAM) size and meristem activity^46^. Similarly, we observed a significant reduction in RAM size of WT due to P_20_ and relatively a larger meristem size in ethylene-insensitive mutants (Fig. 9a b and c). This provides insight into how the primary root length is decreased under excess P_i_ (P_20_). We have also shown that the P_20_ treatment represses the activity of a cell cycle marker Cyclin B1, which is consistent with these RAM size results (Fig. 4a).

In conclusion, we provide comprehensive reference datasets of plant responses under excess and deficient P_i_ regimes. We have demonstrated that the ethylene mediates the negative regulation of plant growth and development like attenuation of primary root length, and decrease in root apical meristem size under excess phosphate. Our study sheds light on the intricate relationship of P, metal ions, ethylene and their effect on plant growth and development. This study not only helps in dissecting the mechanism of plant response to excess P_i_ but also paves the way to identify the pathways and additional novel genes involved in phosphorus homeostasis and accumulation. Although we characterized excess P_i_ responses, furthermore experiments may be required to test the specificity of these responses to other high level nutrient conditions.

## Methods

### Plant materials, growth, and treatments

Wild-type and mutants of *Arabidopsis thaliana* used in the study were in the Columbia (Col) background. Mutant seeds of *etr1–3* (CS3070), *ein4* (CS8053), were obtained from ABRC (Ohio). Seeds of *ein2-T* and *ctr1–1* genotypes were kindly provided by Rishikesh Bhalerao (Umea Plant Science Center, Sweden). Seeds were surface sterilized and suspended in water and incubated 2 days at 4 °C for stratification. Seeds were germinated and grown in 0.5X MS basal medium with vitamins (PhytoTechnology laboratory) + 1.5% (w/v) Sucrose medium on a polypropylene mesh hydroponically for 5 days (Supplementary Fig. S11)^52^. After 5 days, the seedlings were transferred into modified MS nutrient media containing 2.0 mM NH_4_NO_3_, 1.9 mM KNO_3_, 0.15 mM MgSO_4_·7H_2_O, 0.1 mM MnSO_4_·H_2_O, 3.0 µM ZnSO_4_·7H_2_O, 0.1 µM CuSO_4_·5H_2_O, 0.3 mM CaCl_2_.2H_2_O, 5.0 µM KI, 0.1 µM CoCl_2_·6H_2_O, 0.1 mM FeSO_4_·7H_2_O, 0.1 mM Na_2_EDTA.2H_2_O, 1.25 mM KH_2_PO_4_, 100 µM H_3_BO_3_, 1 µM Na_2_MoO_4_·2H_2_O, 1.5% sucrose, 1.25 mM MES, pH 5.7 adjusted with 0.1 M MES (pH 6.1), and grown for 7 days. For P_0_ (0 mM) treatment, KH_2_PO_4_ was replaced with 0.62 mM K_2_SO_4._ For excess phosphate treatments, the concentration of KH_2_PO_4_ was increased in modified MS medium (2.5, 5.0, 10.0, 20.0 mM). We chose the KH_2_PO_4_ salt because potassium is considered as a suitable ion that did not affect the plant growth and development^76–78^. Plants were grown under standard growth condition as 16 h light /8 h dark photoperiod, 120 µmol m^−2^ s^−1^ light intensity, 60–70% humidity, at 23 °C in a growth chamber. Nutrient solutions were changed on alternate days.

### Examination of root system architecture and shoot area

Seedlings were spread gently on agar (1.2%) Petri-plates using art brush. Leaves were dissected and flattened on agar plates. Petri-plates were photographed for the documentation of phenotypic traits. The root length and shoot area were measured using the ImageJ program.

### Anthocyanin measurement

Shoot tissue of Arabidopsis seedlings was separated and ground into powder using liquid N_2_. Around 50–100 mg tissue was used to extract the anthocyanin following the method described by Neff and Chory^79^.

### Estimation of total soluble P_i_ content

Root and shoot tissues were separated, rinsed with distilled water, blotted dry, and ground to fine powder using liquid N_2_. The estimation was carried out using the standard method^31^.

### Histochemical GUS staining

The GUS staining of Arabidopsis genotype *CycB1;1:uidA* ^80^ seedlings was performed as per the standard protocol. The pictures of the stained seedlings were taken in the bright field with a Leica M16 stereomicroscope with an axial carrier using Automontage software (Syncroscopy-Frederick, Maryland USA).

### ICP-OES analysis

ICP-OES analysis was performed as described earlier in our study^53^.

### Quantitative RT-PCR (qRT-PCR)

Total RNA was isolated from frozen root and shoot tissues of 12 days old plant using the method described earlier^81^. The cDNA synthesis and quantitative real-time PCR were carried out as described by Shukla *et al*.^81^. The primer sequences are listed in Supplementary Table S1. The qRT-PCR experiments were repeated two times independently, and in each experiment, three technical replicates were used.

### Transcriptome analysis

Affymetrix GeneChip® ara ST 1.0 GeneChips (data submitted to GEO accession number GSE66925) were used to carry out the microarray experiments following the instructions of the manufacturer (Affymetrix, USA). Three independent biological experiments were carried out for control (1.25 mM P_i_) and experimental conditions (0 mM, 20 mM P_i_) separately for root and shoot tissue of Arabidopsis. The feature intensity (.cel) files were normalized at gene level using RMA algorithms provided in the software Partek Genomics Suite 6.6, St. Louis, MO, USA. To identify statistically significant differentially expressed genes, a two-way ANOVA model was applied to the intensity data. A step-up false discovery rate (FDR) corrected p-value was included for every p-value calculated, and different contrasts were generated. The list of differentially expressed genes (DEGs) was generated by filtering the data at p < 0.1 (FDR) and >2-fold change (P_0_/P_1.25_) and >1-fold change (P_20_/P_1.25_) in expression value.

### GO enrichment analysis of differentially regulated genes

The singular enrichment analysis (SEA) (http://bioinfo.cau.edu.cn/agriGO/index.php) was performed on differentially regulated genes. The TAIR AGI IDs were subjected to agriGO analysis tool using the supported species mode. Arabidopsis TAIR10 database was used as a background. The hieratical graphs and GO enrichment analysis table of genes were constructed, using a Fisher test at P-value 0.05–0.01, with 5 minimum number of mapping entries with complete GO gene ontology. The GO abundance chart was prepared manually by selecting the biologically relevant GO terms.

### Analysis of root apical meristem (RAM)

The mutants and WT seedlings were grown and treated with 1.25 mM P_i_ and 20 mM P_i_ as described in earlier section. The size of the root apical meristem (RAM) was determined by following the protocol of Duan *et al*.^82^. Instead of using Chloral hydrate, we used Visikol^Tm^ (Visikol Inc.) for clearing the root section. Visikol was diluted 200 times in 10% glycerol and applied to the root tissue. The number of cells counted between the quiescent cells and to the first elongated cells of the meristem was visualized with Carl Zeiss Axioplan 2 Imaging fluorescent microscope at 20X magnification using Differential Interference Contrast (DIC) filter.

### Statistics

Each experiment was repeated 2–3 times independently. Statistical significance of mean values was determined by analysis of variance (1X-, 2X-ANOVA) with Tukey’s Multiple Comparison Test or Bonferroni correction post-test were used for comparing all pairs of data sets.

## Electronic supplementary material

Supplementary information

Dataset 1

Dataset 2

Dataset 3

Dataset 4
