# Supplementary material for: Comprehensive study of excess phosphate response reveals ethylene mediated signaling that negatively regulates plant growth and development
Source: Sci Rep. 2017 Jun 8;7:3074. doi: 10.1038/s41598-017-03061-9 (PMC5465178; doi:10.1038/s41598-017-03061-9)
Supplement: Supplementary file 1 — Supplementary information [file 41598_2017_3061_MOESM1_ESM.pdf]

ONLINE DATA SUPPLEMENT

Comprehensive study of excess phosphate response reveals ethylene mediated signaling that negatively regulates plant growth and development

Devesh Shukla*, Claire A. Rinehart, Shivendra V. Sahi*

Department of Biology, 1906 College Heights, Western Kentucky University, Bowling Green, 42101-1080, Kentucky, U.S.A.

*****Authors for correspondence

Email address: shiv.sahi@wku.edu
Tel: +1-270-745-6012, Fax: +1-270-745-6856

Email address: devesh.shukla@wku.edu
Tel: +1-270-745-2389, Fax: +1-270-745-6856

Supplementary Dataset File 1. RMA Log Gene Level 2-wayANOVA Results

Supplementary Dataset File 2. Differentially expressed genes in root

Supplementary Dataset File 3. List of significant enriched GO terms

Supplementary Dataset File 4. Differentially expressed genes in shoot

Supplementary Figure S1. Supply of different concentrations of excess phosphate (P_i) represses the expression of high-affinity phosphate uptake transporters.

Supplementary Figure S2. Supply of different concentrations of excess phosphate (P_i) modulates the expression of phosphate xylem loading transporter (PHO1) and P_i starvation inducible gene (RNS1).

Supplementary Figure S3. The Principal Component Analysis (PCA) analysis of whole transcriptome of Arabidopsis root and shoot under different P_i treatment.

Supplementary Figure S4. Molecular function hieratical graph generated using Singular Enrichment Analysis of differentially expressed unique genes of root due to P20 treatment.

Supplementary Figure S5. Biological process hieratical graph generated using Singular Enrichment Analysis of differentially expressed commonly regulated genes of shoot due to treatments P_0, P_{20}.

Supplementary Figure S6. Biological Process hieratical graph generated using Singular Enrichment Analysis of differentially expressed unique genes of shoot due to P_{20} treatment.

Supplementary Figure S7. Biological process hieratical graph generated using Singular Enrichment Analysis of differentially expressed genes of root+shoot due to treatments P_{20}.

Supplementary Figure S8. Quantitative real-time PCR validation of the microarray expression data.

Supplementary Figure S9. Effect of excess phosphate supply on root apical meristem size of ethylene- insensitive mutants (*etr1-3, ein4*) indicating the involvement of ethylene signaling.

Supplementary Figure S10. Effect of excess phosphate supply on root apical meristem size of sensitive mutants (*ctr1-1*) indicating the involvement of ethylene signaling.

Supplementary Figure S11. Pictorial depiction of components of hydroponic setup.

Supplementary Table S1. List of primers used for qRT-PCR

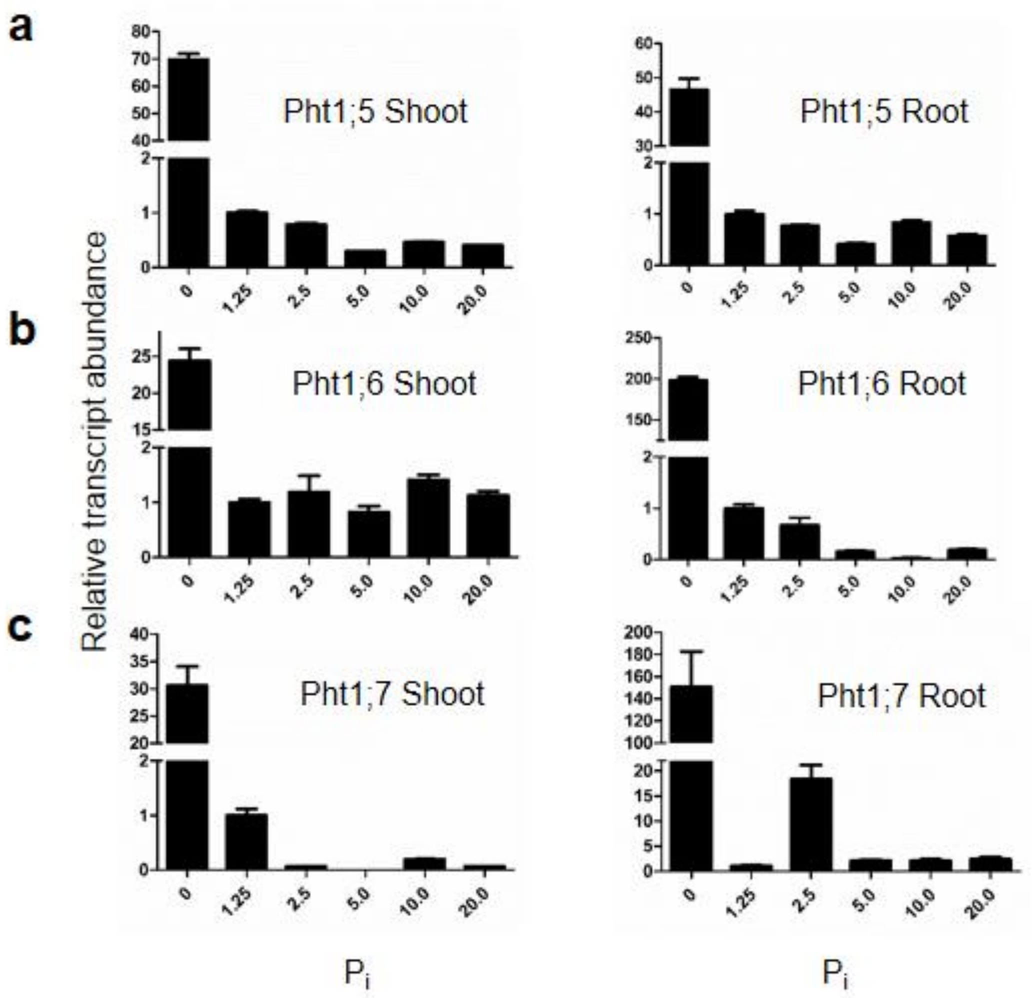

Supplementary Figure S1. Supply of different concentrations of excess phosphate (P_i) represses the expression of high-affinity phosphate uptake transporters. Quantitative real-time PCR analysis of expression level of Pht1;5 (a), Pht1;6 (b), and Pht1;7 (c) in the roots and shoots of WT seedlings treated with different concentrations of excess phosphate, as described in the legend to Figure 1. Beta-tubulin was used as an internal control. Data are expressed as the mean of three biological replicates with three technical replicates.

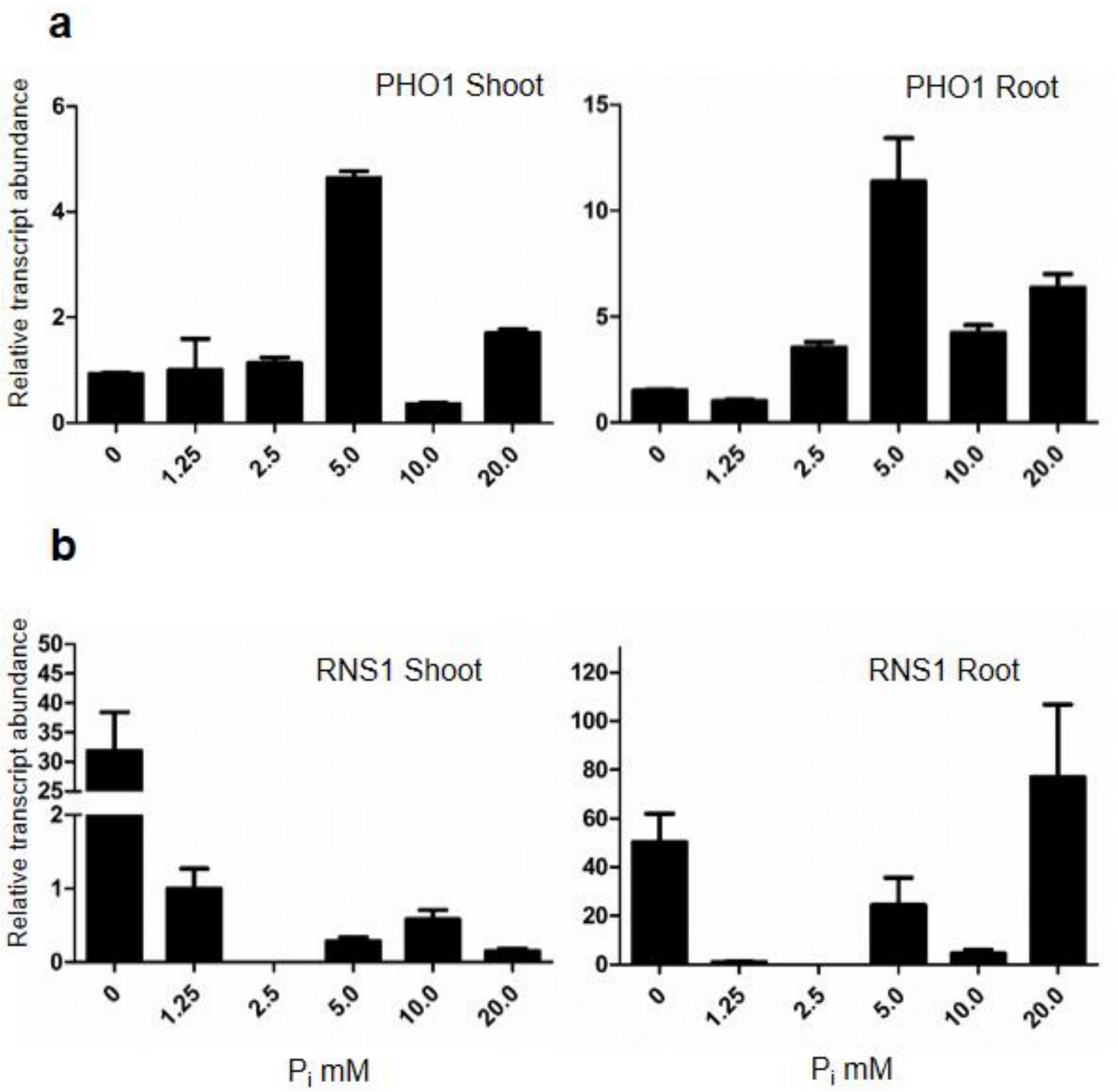

Supplementary Figure S2. Supply of different concentrations of excess phosphate (P_i) modulates the expression of phosphate xylem loading transporter (PHO1) and P_i starvation inducible gene (RNS1). Quantitative real-time PCR analysis of expression level of PHO1 (a) and RNS1 (b) in the roots and shoots of WT seedlings treated with different concentrations of excess phosphate, as described in the legend to Figure 1. Beta-tubulin was used as an internal control. Data are expressed as the mean of three biological replicates with three technical replicates.

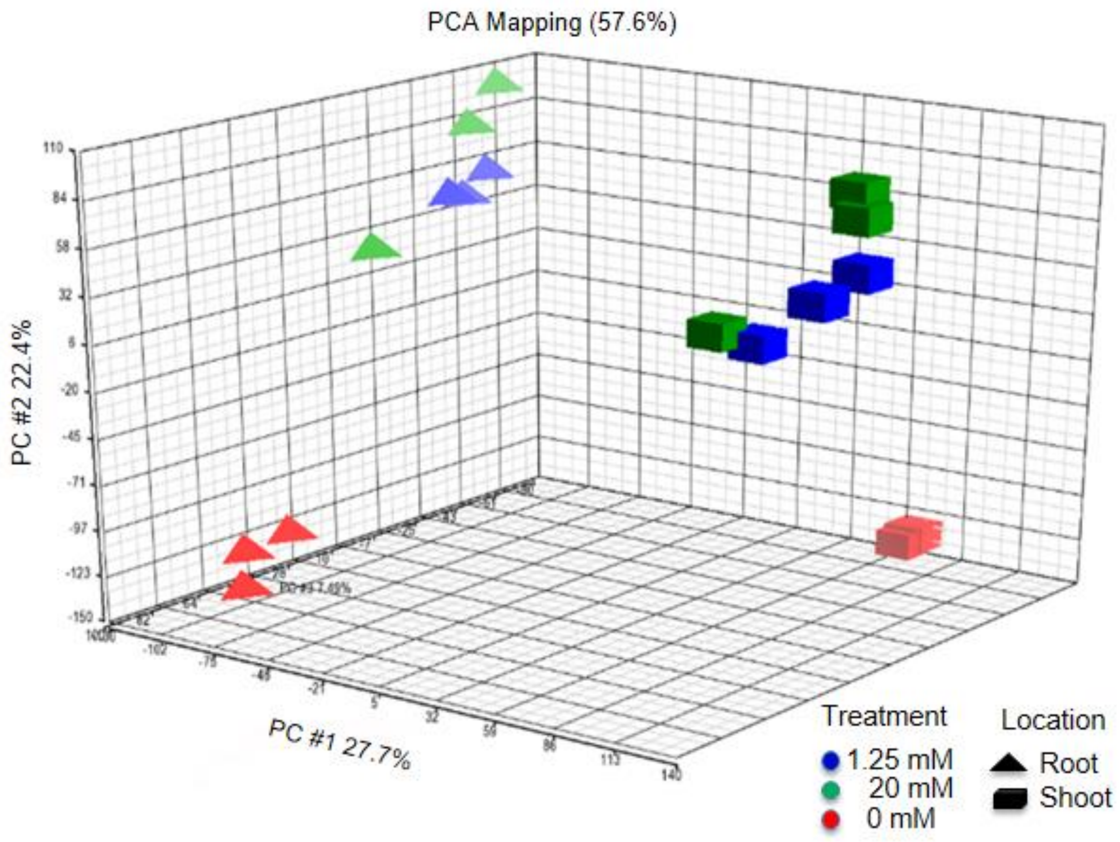

Supplementary Figure S3. The Principal Component Analysis (PCA) of whole transcriptome of Arabidopsis root and shoot under different P_i treatment. It shows remarkable differences between the root and shoot across the control and treated samples.

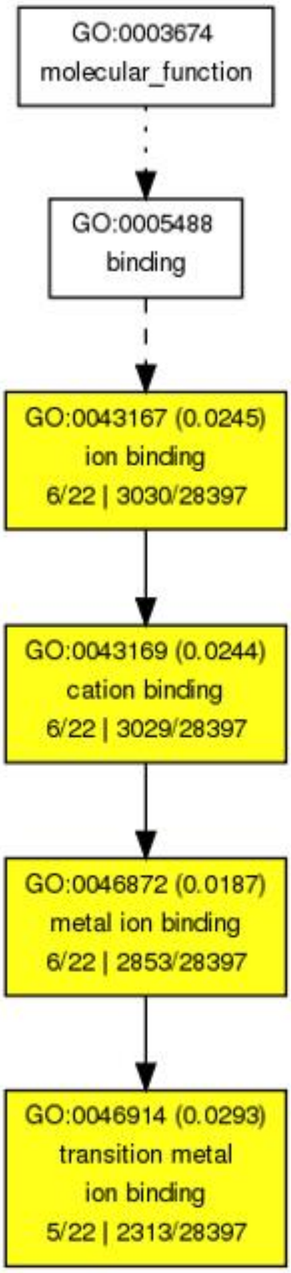

Supplementary Figure S4: Molecular function hieratical graph generated using Singular Enrichment Analysis of differentially expressed unique genes of root due to P_{20} treatment. The analysis was performed using agriGO tool under advanced mode, Fisher test with P< 0.05 and minimum 5 mapping entries.

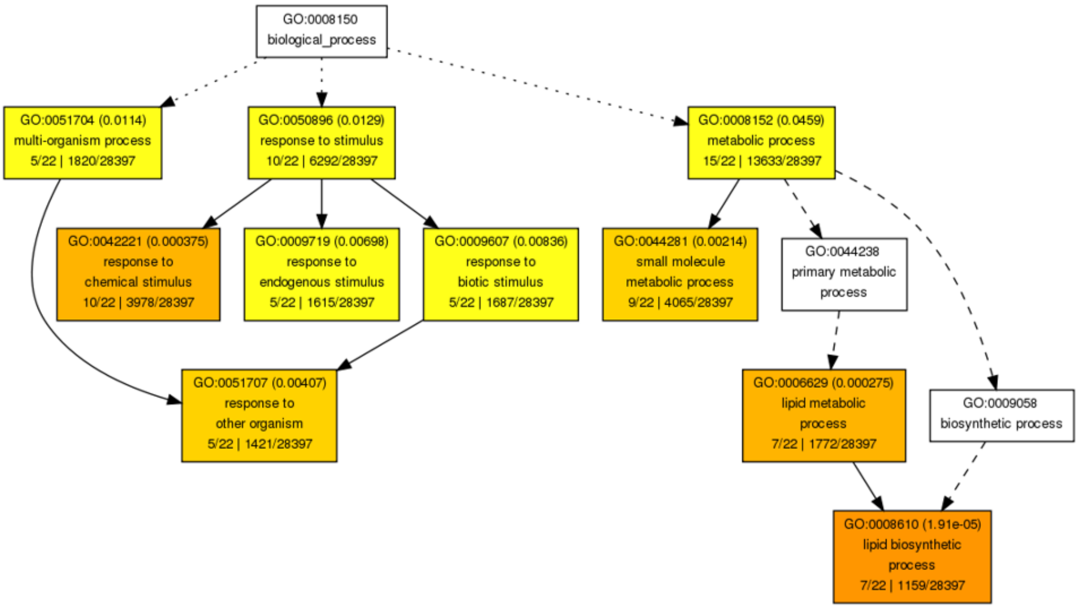

Supplementary Figure S5: Biological process hieratical graph generated using Singular Enrichment Analysis of different expressed commonly regulated genes of shoot due to treatments P_0, P_{20}. The analysis was performed using agriGO tool under advanced mode, Fisher test with P<0.05 and minimum 5 mapping entries.

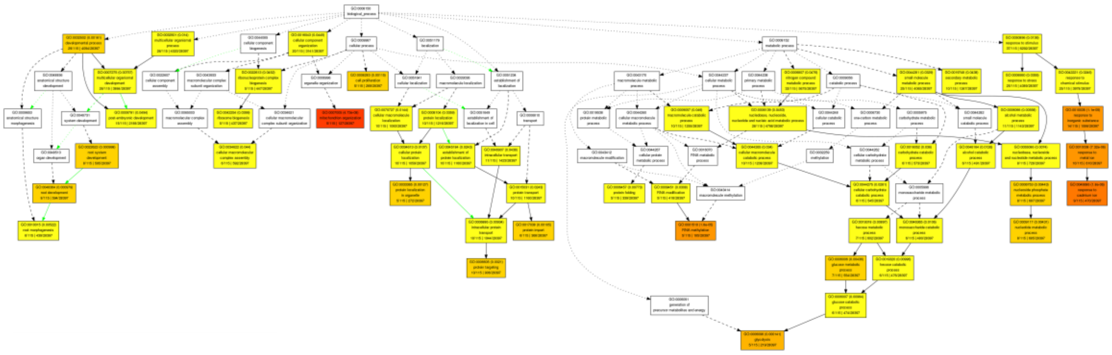

Supplementary Figure S6: Biological process hieratical graph generated using Singular Enrichment Analysis of differentially expressed unique genes of shoot due to P_{20} treatment. The analysis was performed using agriGO tool under advanced mode, Fisher test with P<0.01 and minimum 5 mapping entries.

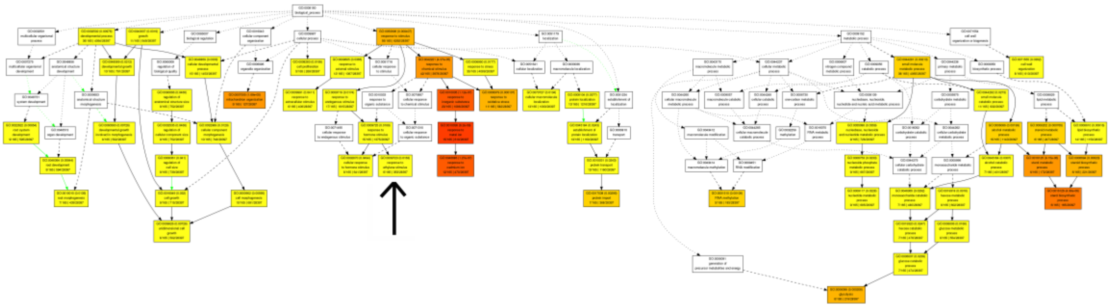

Supplementary Figure S7: Biological process hieratical graph generated using Singular Enrichment Analysis of differentially expressed genes of root+shoot due to P_{20} treatment. The analysis was performed using agriGO tool under advanced mode, Fisher test with $P<0.05$ and minimum 5 mapping entries. Arrow mark in the figure indicates ethylene response GO term.

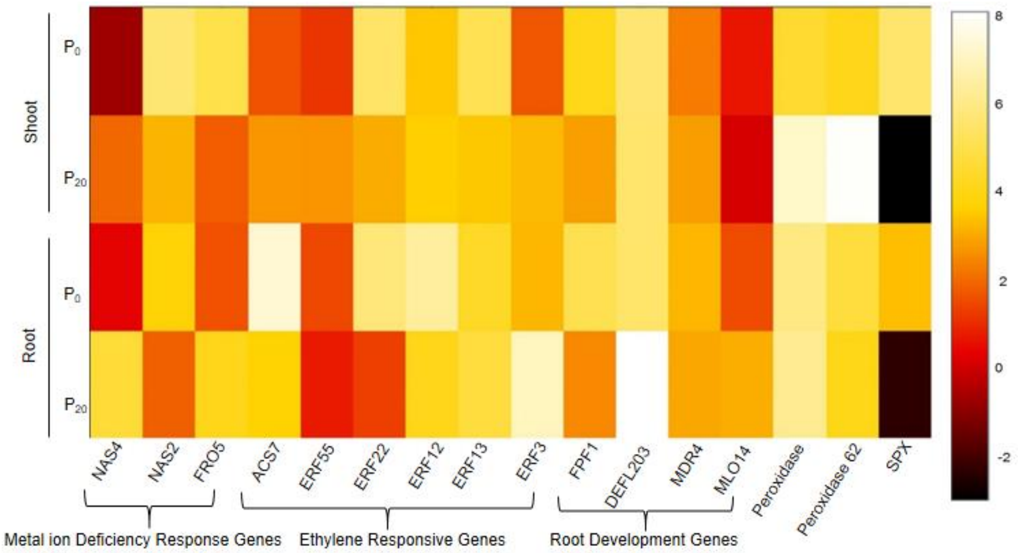

Supplementary Figure S8: Quantitative real-time PCR validation of the microarray expression data. These results show the by-enlarge positive correlation with the microarray expression data. The qRT-PCR was carried out for the root and shoot tissues of Arabidopsis treated and grown in similar condition in which microarray was performed. The real time data have been analyzed using the relative quantification (ddct) method and values have been log2 transformed. The number/colors of the scale show the log2 transformed fold-change values as detected by qRT-PCR compared to $P_{1.25}$. This heat map has been constructed using “plotly”, an online plot tool.

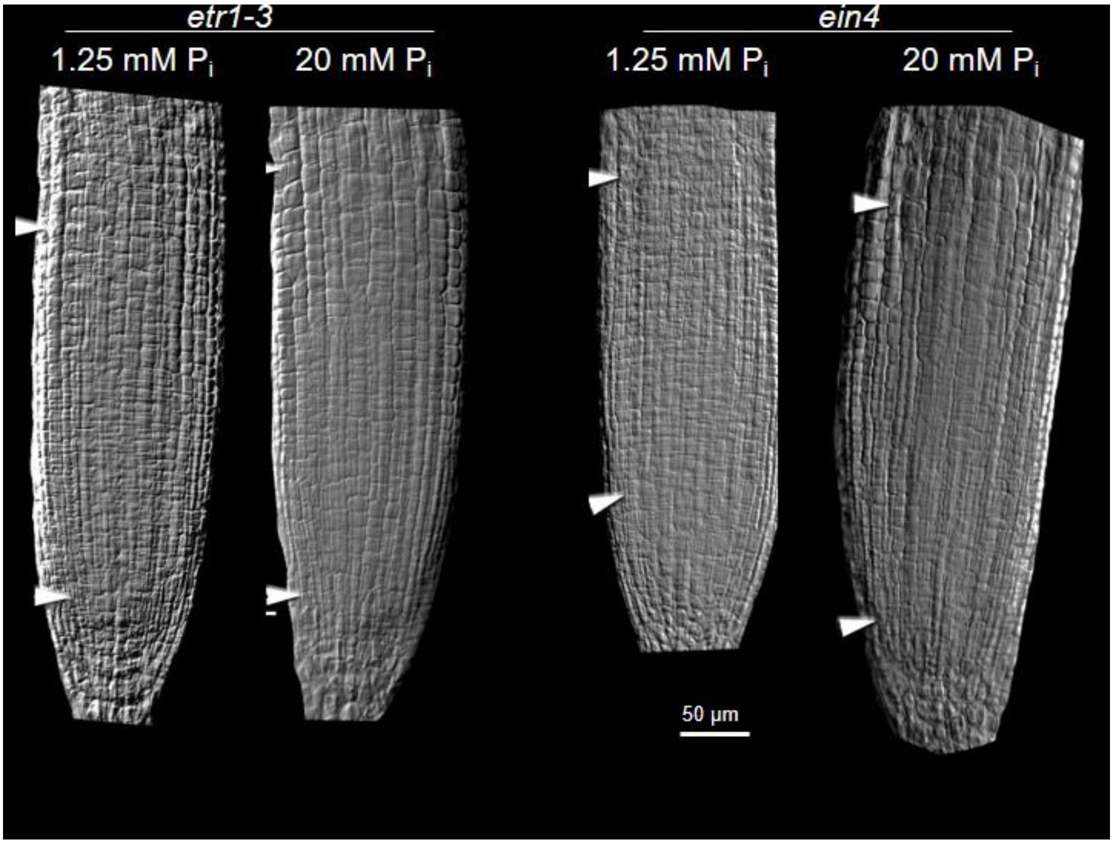

Supplementary Figure S9. Effect of excess phosphate supply on root apical meristem size of ethylene- insensitive mutants (*etr1-3, ein4*) indicating the involvement of ethylene signaling. Mutants (*etr1-3, ein4*) were grown as described in the caption to Figure 1 a, and processed as described in the caption to Figure 8.

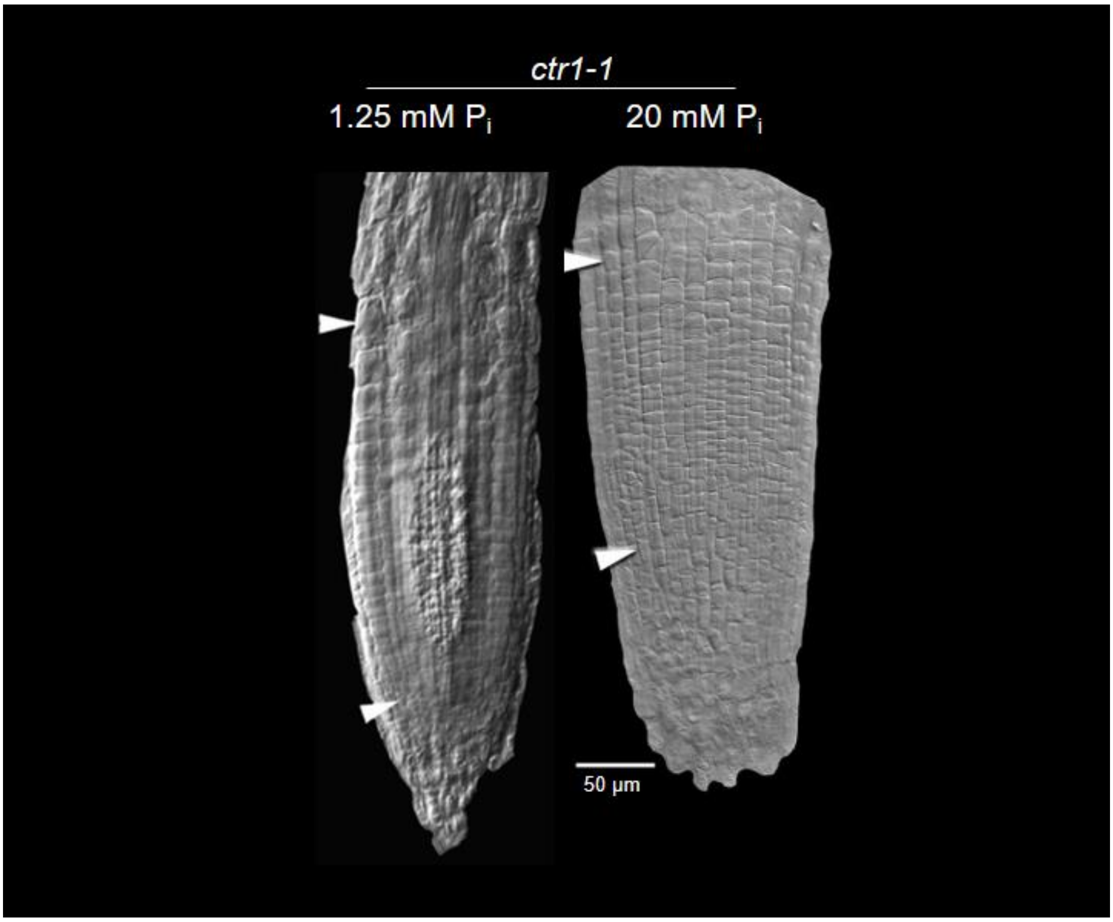

Supplementary Figure S10. Effect of excess phosphate supply on root apical meristem size of sensitive mutants (*ctr1-1*) indicating the involvement of ethylene signaling. Mutant *ctr1-1* were grown as described in the caption to Figure 1 A, and processed as described in the caption to Figure 8.

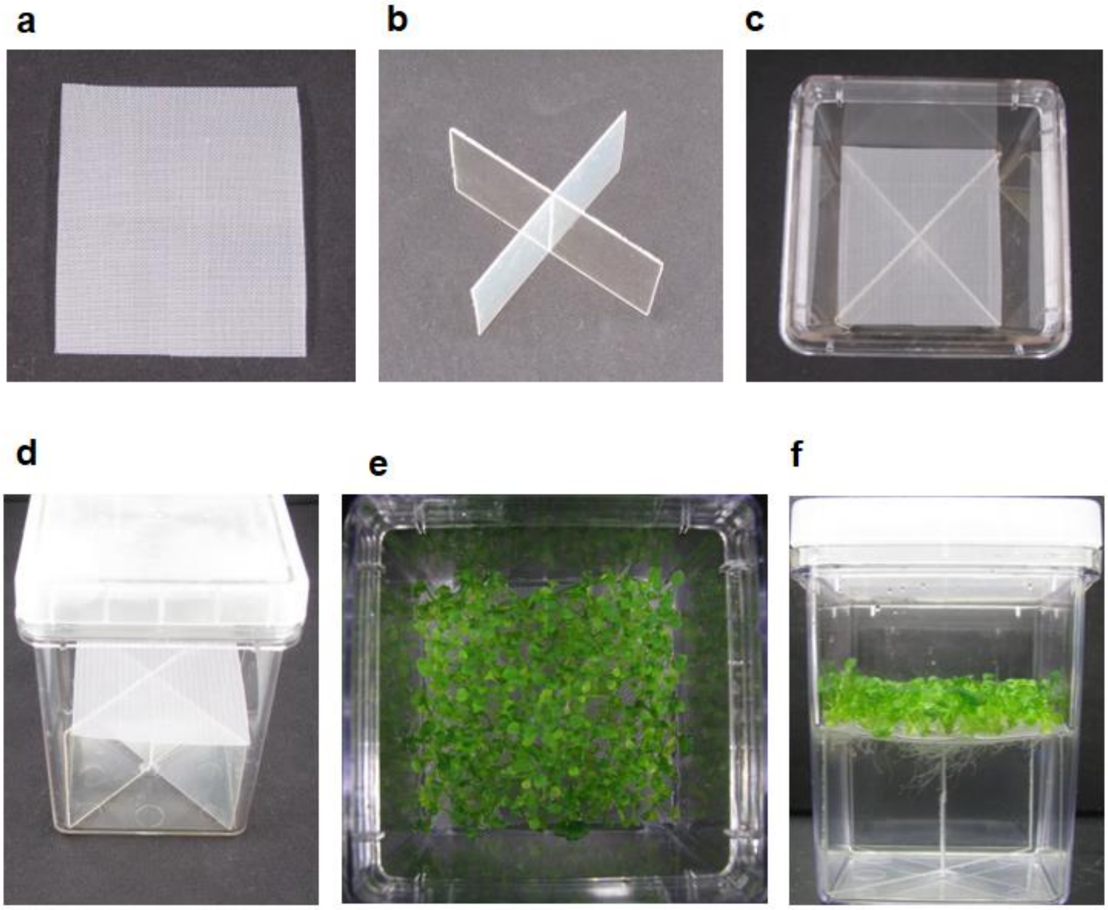

Supplementary Figure S11. Pictorial depiction of components of hydroponic setup. A polypropylene mesh (a), a rectangular pieces polycarbonate (b), standard magenta box (c), assembly of the mesh in the magenta box (d), top view of Arabidopsis seedlings grown on the mesh (e), side view of the Arabidopsis seedlings grown in the magenta box (f).

Supplementary Table S1: List of primers used for qRT-PCR in this study

Sequence 5' to 3'	Gene
ATC GTC CCG TTA AAC GAC TCC	AtSPX F
CTC CAT TGA ATC CTT AGC TTT CGC	AtSPX R
GCT GTT TAT ACG GCA AGA CAC C	AtMDR4 F
CTC CAT TTA AGC CGC TCT CTG A	AtMDR4 R
CTA ACG ACG CCC TTC TAG TTC C	AtACS7 F
GTC GCA GTG GAT GGG TAC TAT T	AtACS7 R
TCT CCA AGT GAC TCT CTA GCC A	At Peroxidase 62 F
TCC GCT CGA GTT GAA TAT CCT G	At Peroxidase 62 R
CCA GTT GTC CTA CAG AGG CAT T	AtERF022 F
GAC GAG TTC CGG AAA GTT GAG A	AtERF022 R
GTT TAA GCT TCG TGG TGA CTC G	AtERF55 F
ACC ATA TTC GCC GGT ATC AGA C	AtERF55 R
CAG CTG CTA GAG CTT ACG ATG T	AtERF012 F
CTC TTT GGA TGG ATT TGG GGG A	AtERF012 R
CGC TAA CTC CTC TTC TGA CCA T	AtERF013 F
AGA AGC AGA TGG ATG GTG ATC G	AtERF013 R
CAT TCA CAC TAA TGG CAC GAC C	AtERF003 F
CTC AAA CGT CCC TAG CCA GAT T	AtERF003 R
TCA AGG ACG TTG TTG TCC TCT C	At Peroxidase F
AGA GTC TGA GGA GTC CAC GTT A	At Peroxidase R
GAC TTG TGG AGA ACC CTA ACC A	AtFPF1 F
GAC CAC TTC TCC TGT CGG TAA A	AtFPF1 R
ACA TAT GGA CCC CCA AAC TCG C	AtFRO5 F
CCC ATT CTC CCT TTT ACC TTC CA	AtFRO5 R
TTC TAA TGG ACT CCC AAA GGC G	AtDEFL203 F
TAA CAA GGT CCG TAG CAG TGA C	AtDEFL203 R
GTT AAT TCG GTG GTC ATC GCA C	AtNAS2 F
GAT TGT TCA AGA TCG CGT GGA C	AtNAS2 R
GGG GCA TGA GCC ATT TCT TTC	AtMLO14 F
TTC GCG TGC AAC CAC AGT TA	AtMLO14 R
GTT AGA GGA GAA CGA GCA TGA CCA	AtPht1;8 F
CAT CGT GTC TGA AGC AAA GTG CTC	AtPht1;8 R
CCG GAG TCT AAA GGG AAG TCT TTG	AtPht1;5 F
TTG CCA CAG TCA TCT CCA CCAC	AtPht1;5 R
GCT TGG CTT GCT TCT CAC GTT TA	AtPht1;6 F
CCT TGA TTT TCT CCG GCT CAG TT	AtPht1;6 R
TGG TTC CTG AAT CTA AAG GCA AGT C	AtPht1;7 F
TCT ATG CAG TTG ACA CTG CGT TGTT	AtPht1;7 R
GGA GGG TTT AAG GAT CGA ACC AAG	AtPHO1 F
GTC TTC TTC GTT TTG CAC TTT GGAGCG	AtPHO1 R
CCA CAT GGG AAA TGT GGA GCT GAG	AtRNS1 F
GAT CGA TGC CGG TTC AAG AGA CTG A	AtRNS1 R

CTCACAGTCCCGGAGCTGACAC	At Tubulin beta F
GCTTCAGTGAACTCCATCTCGT	At Tubulin beta R
